# Uncleavable fusion of Csy4 with prime editors for low Csy4 toxicity and highly efficient prime editing in Arabidopsis

**DOI:** 10.1093/plphys/kiag262

**Published:** 2026-05-18

**Authors:** Wei Sun, Yu Lu, Zhenghong Cao, Cuiping Xin, Kexin Fan, Xiangchao Kong, Xiaolong Chen, Mingyu Zhang, Qi-Jun Chen

**Affiliations:** State Key Laboratory of Plant Environmental Resilience, College of Biological Sciences, China Agricultural University, Beijing 100193, China; State Key Laboratory of Plant Environmental Resilience, College of Biological Sciences, China Agricultural University, Beijing 100193, China; State Key Laboratory of Plant Environmental Resilience, College of Biological Sciences, China Agricultural University, Beijing 100193, China; State Key Laboratory of Plant Environmental Resilience, College of Biological Sciences, China Agricultural University, Beijing 100193, China; State Key Laboratory of Plant Environmental Resilience, College of Biological Sciences, China Agricultural University, Beijing 100193, China; State Key Laboratory of Plant Environmental Resilience, College of Biological Sciences, China Agricultural University, Beijing 100193, China; State Key Laboratory of Plant Environmental Resilience, College of Biological Sciences, China Agricultural University, Beijing 100193, China; State Key Laboratory of Plant Environmental Resilience, College of Biological Sciences, China Agricultural University, Beijing 100193, China; State Key Laboratory of Plant Environmental Resilience, College of Biological Sciences, China Agricultural University, Beijing 100193, China; Center for Crop Functional Genomics and Molecular Breeding, China Agricultural University, Beijing 100193, China

## Abstract

An uncleavable fusion of prime editors with the Csy4 CRISPR RNA endonuclease efficiently generates heritable mutations in Arabidopsis with reduced Csy4-associated toxicity.

Dear Editor,

Prime editing enables precise genetic modifications, including base substitutions, insertions, and deletions, at targeted genomic loci (Anzalone et al. 2019). Although prime editors (PEs) have been successfully established in monocots (Cao et al. 2024; Lu et al. 2026), overcoming the efficiency barrier in Arabidopsis and other dicots remains challenging. Several optimization strategies—such as fusing an RNA chaperone to PEmax along with heat treatment (Vu et al. 2024) and using split PE proteins (Lu et al. 2025)—have improved prime-editing efficiency in Arabidopsis; however, none have yielded homozygous or heterozygous mutants in T1 plants. Inhibition of RDR6 markedly enhances prime-editing performance in Arabidopsis, yet the highest efficiencies of the optimized PEs for generating homozygous or heterozygous mutants are only 1.4% (1/70, 47.4% desired reads), 1.9% (1/53, 46.1% desired reads), 2.2% (1/45, 50.9% desired reads), and 1.9% (1/53, 63.2% desired reads) at the *GL1*, *SOV*, *URT1-m1.2*, and *URT1-m2* loci, respectively (Ren et al. 2025). In our previous work, we demonstrated that PE6c and Csy4-based PEs greatly enhance prime-editing efficiency in rice (Cao et al. 2024; Lu et al. 2026). The sufficient protection of the 3′ end of pegRNAs by the pegRNA-Csy4RS-Csy4 complex accounts for the high-efficiency of Csy4-based PEs (Lu et al. 2026). We reasoned that Csy4-based PEs would similarly improve editing efficiency in Arabidopsis. In addition, as the only research group to report strong Csy4 toxicity in plants including maize and Arabidopsis (Jiang et al. 2020), we have maintained close attention to the toxicity of Csy4-based PEs. We therefore sought to determine whether uncleavable Csy4-PEs could help mitigate the toxicity associated with cleavable Csy4-P2A-PEs. Here, we demonstrate that fusing Csy4 with prime editors enables highly efficient prime editing in Arabidopsis. In addition, we revealed that cleavable Csy4-P2A-PE fusions have severely negative effects on Arabidopsis transformations, while uncleavable Csy4-PE fusions mitigate the intrinsic toxicity of Csy4.

We generated 6 types of Csy4-based PEs, using PEmax, PE6c, or PE6d, which can be classified into 2 categories: cleavable Csy4-P2A-PEs, in which Csy4 and PEs are separated by the P2A peptide, and uncleavable Csy4-PEs (Fig. 1a; Supplementary Methods; Supplementary Sequences S1–S6). We constructed 3 RNA cassettes to express epegRNAs and sgRNAs flanked by the Csy4 recognition site (Csy4RS) (Fig. 1b; Supplementary Methods; Supplementary Sequences S7–S9; Tables S1 and S2). RNA cassette V2 was designed to increase pegRNA expression relative to cassette V1, and cassette V1×2 was generated to support the expression of duplex PEs (Fig. 1b). In total, we assembled 42 binary vectors: 24 vectors each carrying one of the 3 Csy4-P2A-PE types with RNA cassette V1 or V2 across 4 targets (Figure S1); 12 vectors each carrying 1 of the 3 Csy4-PE types with RNA cassette V2; and 6 vectors each carrying 1 of the 3 duplex Csy4-PEs. These constructs were introduced into Arabidopsis using the floral dip method.

**Figure 1:**
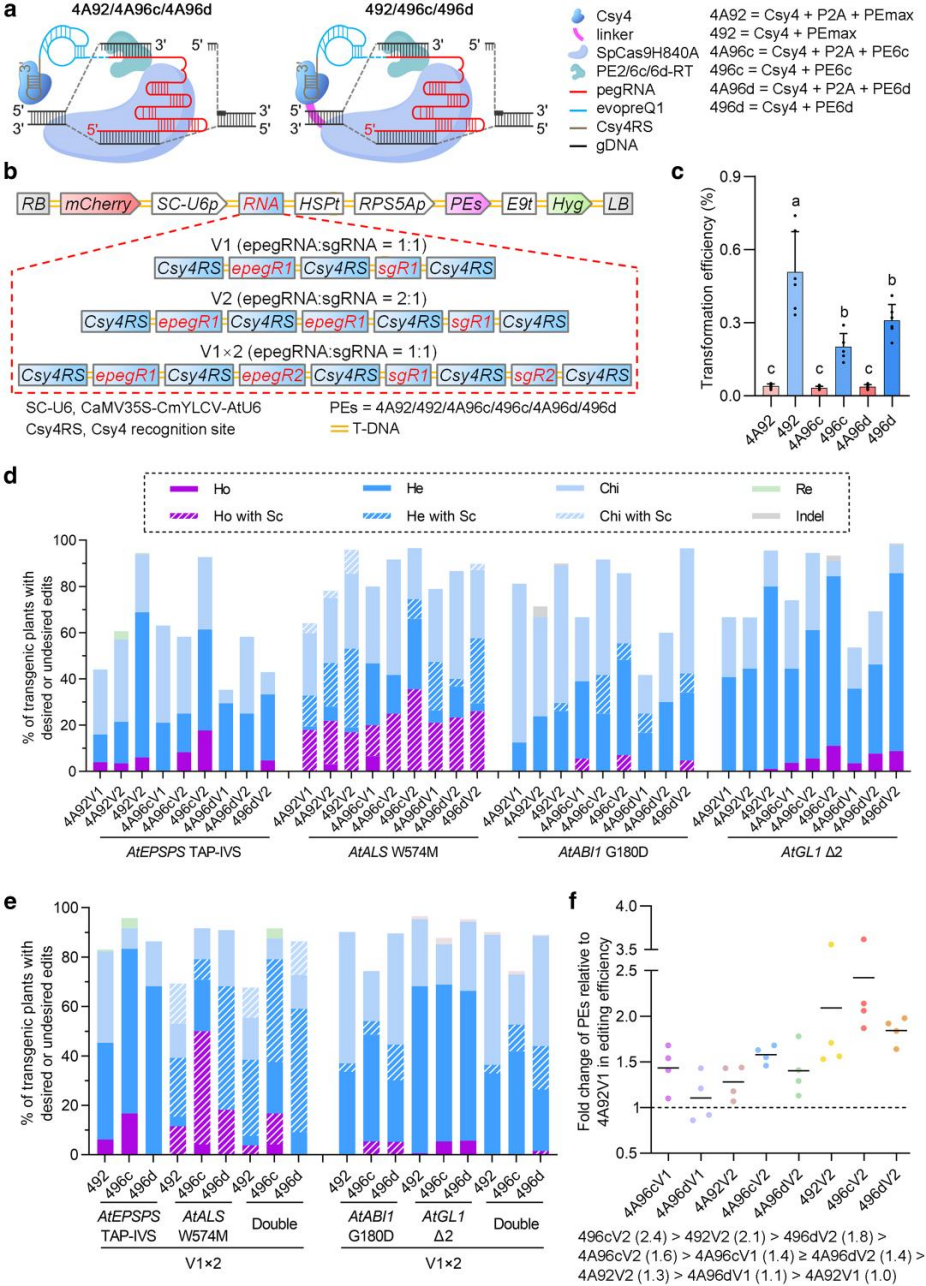
Uncleavable fusion of Csy4 with prime editors overcomes the intrinsic toxicity of Csy4 and enables high-efficiency prime editing in Arabidopsis. a) Schematic diagram of 6 types of Csy4-based prime editors, which are classified into 2 groups: cleavable Csy4-P2A-PEs (left) and uncleavable Csy4-PEs (right). b) Schematic representation of the T-DNA constructs used to generate transgenic Arabidopsis, involving 6 types of PE proteins and 3 RNA cassettes expressing pegRNAs and sgRNAs. c) Transformation efficiencies of the 6 types of PEs in combination with 3 RNA cassettes across 4 targets are shown. 4A9* (* = 2, 6c, or 6d), 4A9*V1 or V2; 49*, 49*V2 or V1×2. A total of 24 and 12 transformations with 4A9*V1/V2 and 49*V2, respectively, across 4 targets were performed; and 6 additional transformations using 49*V1×2 duplex PEs were performed. One-way ANOVA followed by Tukey's multiple-comparisons test was used; bars sharing a lowercase letter indicate no significant difference (*P* > 0.05). d) Sorting-based editing efficiencies of 6 types of PEs along with RNA cassette V1, V2, or both. Ho, He, and Chi are homozygous, heterozygous, and chimeric mutant lines, respectively. Re, DNA repair-derived byproducts with only some of the target bases edited when installing multiple-base substitution edits. Sc, pegRNA scaffold-derived byproducts. Ho or He with Sc, homozygous or heterozygous mutant lines harboring desired edits along with the pegRNA scaffold-derived mutations. e) Sorting-based editing efficiencies of 6 duplex PEs. f) Average fold change in prime editing efficiency across the 4 target sites. Points, horizontal lines, and the dashed line represent values at each target, the mean across the 4 targets, and the value for 4A92V1, respectively.

Because intrinsic Csy4 toxicity has been observed in human cells (Nissim et al. 2014) and in plants including Arabidopsis (Jiang et al. 2020), we first assessed the effects of cleavable and uncleavable Csy4 on the 42 Arabidopsis transformations. All 24 vectors harboring cleavable Csy4-P2A-PEs exhibited strong toxic effects, resulting in an average transformation efficiency of only 0.037% (Fig. 1c; Table S3). By contrast, the 18 vectors harboring uncleavable Csy4-PEs showed largely normal transformation efficiencies, averaging 0.34%; notably, the transformation efficiency of Csy4-PEmax (492) increased by 13.5-fold relative to Csy4-P2A-PEmax (4A92) (Fig. 1c; Table S3). These findings indicate that uncleavable fusion of Csy4 with prime editors largely mitigates Csy4-associated toxicity. Six transformations using Csy4-PEmax (492V2/V1×2) yielded an average efficiency of 0.51%, which was substantially higher than that obtained with Csy4-PE6c (496cV2/V1×2, 0.20%) or Csy4-PE6d (496dV2/V1×2, 0.30%), suggesting residual weak toxicity of Csy4-PE6c and Csy4-PE6d in Arabidopsis (Fig. 1c; Table S3). Because transformation efficiencies varied considerably among experiments, more stringent and standardized conditions along with non Csy4-based PEs as controls will be required in future studies to more accurately evaluate transformation efficiency and, consequently, the residual weak toxicity of uncleavable Csy4-PE fusions.

We analyzed the editing efficiencies of the 6 Csy4-based PEs along with the 2 RNA cassettes across 4 targets in Arabidopsis using a sorting-based assay (Fig. 1d; Table S4). All 6 Csy4-based PEs exhibited high prime-editing efficiencies across all targets (Fig. 1d; Table S4). In T1 plants, the frequencies of homozygous and heterozygous mutations ranged from 16.0% to 68.8%, 32.8% to 74.6%, 12.5% to 55.4%, and 35.7% to 85.7% at the *EPSPS*, *ALS*, *ABI1*, and *GL1* loci, respectively (Fig. 1d; Table S4). Sanger sequencing of representative lines confirmed the consistency of the sorting-based assay results (Figure S2). The 6 duplex PEs also produced high prime-editing efficiencies, generating homozygous or heterozygous mutations in 45.4% to 83.3% (*EPSPS*), 39.2% to 79.2% (*ALS*), and 38.5% to 79.2% (combined targets), 37.0% to 54.1% (*ABI1*), 66.3% to 68.9% (*GL1*), and 36.4% to 52.7% (combined targets) of transgenic plants (Fig. 1e; Table S4).

To compare the editing efficiencies of the 6 PEs in combination with the 2 RNA cassettes, we performed reads-based analysis of prime-editing activity (Cao et al. 2024; Lu et al. 2026) (Fig. 1f; Figure S3; Tables S5–S10). The fold improvements relative to the 4A92V1 control were 2.4 (496cV2), 2.1 (492V2), 1.8 (496dV2), 1.6 (4A96cV2), 1.4 (4A96cV1), 1.4 (4A96dV2), 1.3 (4A92V2), and 1.1 (4A96dV1) (Fig. 1f). Editing efficiencies ranked as follows: (a) 492V2 (2.1) >4A92V2 (1.3) >4A92V1 (1.0); (b) 496cV2 (2.4) >4A96cV2 (1.6) >4A96cV1 (1.4); (c) 496dV2 (1.8) >4A96dV2 (1.4) >4A96dV1 (1.1); (d) 4A96cV1 (1.4) >4A96dV1 (1.1) >4A92V1 (1.0); (e) 4A96cV2 (1.6) >4A96dV2 (1.4) >4A92V2 (1.3); (f) 496cV2 (2.4) >492V2 (2.1) >496dV2 (1.8). These results demonstrate that uncleavable Csy4-PEs outperform cleavable Csy4-P2A-PEs, that enhanced epegRNA expression (V2 versus V1) increases editing efficiency, and that Csy4-based PE6c systems outperform the corresponding PE6d and PEmax systems.

To assess the inheritance of mutations in the prime-edited lines, we isolated T-DNA-free T2 seeds from T1 transgenic lines identified as homozygous mutants. As expected, the sorting-based assay confirmed that all T-DNA-free T2 plants were homozygous for the edited alleles (Table S11). Phenotypic analysis of representative T2 mutants showed that the *ALS*-W574M and *GL1*-Δ2 mutations conferred the expected phenotypes. Interestingly, *EPSPS* TAP-IVS mutant seedlings displayed glyphosate resistance in leaves but not in roots, suggesting that the expression level of *EPSPS* in seedling roots is substantially lower than that in leaves in Arabidopsis (Figure S4).

Our results revealed that high-frequency byproducts derived from the pegRNA scaffold (Sc) were generated at the *ALS* target site (Fig. 1d and e). This observation is consistent with previous reports showing that enhanced prime editing efficiency is often accompanied by increased Sc byproduct formation (Jiang et al. 2022; Qiao et al. 2023). These unwanted editing outcomes can be effectively minimized through careful design of the reverse transcriptase template (rtT), following 2 established principles. First, the 5′ end of the rtT should terminate immediately after a C, GC, or TGC motif in the genomic DNA, corresponding to the 3′ end of the sgRNA scaffold (Jiang et al. 2020, 2022). Alternatively, the rtT can be designed such that erroneous substitutions involving C or G—which account for the majority of Sc byproducts—result only in silent mutations (Qiao et al. 2023). We redesigned the rtT for the ALS-W574 mutation and successfully eliminated Sc byproducts while maintaining high editing efficiency (Figure S5; Table S12).

In conclusion, we developed a toolkit for highly efficient prime editing and minimal Csy4 toxicity in Arabidopsis through the use of uncleavable Csy4-PE fusions. We quantified the extent of Csy4-associated toxicity during Arabidopsis transformation, and our findings provide a strong foundation for developing Csy4-based prime-editing systems in other dicot species, particularly when combined with additional strategies such as geminiviral replicon systems, where the increased Csy4 toxicity resulting from high expression levels of cleavable Csy4-P2A-PE fusions could pose a major challenge.

## Supplementary Material

kiag262_Supplementary_Data

## Data Availability

The data underlying this article will be shared on reasonable request to the corresponding author.
