# Supplementary material for: Uncleavable fusion of Csy4 with prime editors for low Csy4 toxicity and highly efficient prime editing in Arabidopsis
Source: Plant Physiol. 2026 May 18;201(1):kiag262. doi: 10.1093/plphys/kiag262 (PMC13181393; doi:10.1093/plphys/kiag262)
Supplement: kiag262_Supplementary_Data [file kiag262_supplementary_data.zip › Sun et al. PLPHYS-2025-2311R2 Suppl Data.pdf]

Supplementary Data

Uncleavable fusion of Csy4 with prime editors for low Csy4 toxicity and highly efficient prime editing in Arabidopsis

Wei Sun[1,†], Yu Lu[1,†], Zhenghong Cao[1,†], Cuiping Xin[1], Kexin Fan[1], Xiangchao Kong[1], Xiaolong Chen[1], Mingyu Zhang[1], Qi-Jun Chen[1,2,*]

[1]State Key Laboratory of Plant Environmental Resilience, College of Biological Sciences, China Agricultural University, Beijing 100193, China

[2]Center for Crop Functional Genomics and Molecular Breeding, China Agricultural University, Beijing 100193, China

Author for correspondence: Qi-Jun Chen, qjchen@cau.edu.cn

[†]These authors contributed equally to this work.

Table of contents

Supplementary Figure S1. Annotated genomic DNA sequences... ...and nicking sgRNAs 3
Supplementary Figure S2. Representative sequencing chromatograms ... ...Sc byproducts..................... 4
Supplementary Figure S3. Reads-based editing efficiencies… ...across four targets 5
Supplementary Figure S4. Phenotypes of T2 plants from T1 homozygous mutants. 7
Supplementary Figure S5. Redesign of rtT of pegRNA... ...eliminated the Sc byproducts......................... 8
Supplementary Table S1. Sequences related to pegRNAs and nicking sgRNAs 9
Supplementary Table S2. Sequences of primers... 10
Supplementary Table S3. Transformation efficiencies of 24… ...harboring uncleavable Csy4-PEs..........11
Supplementary Table S4. Mutation efficiencies... ... in Arabidopsis 12
Supplementary Table S5. Percentage of desired reads... ...targeting *EPSPS*... 12
Supplementary Table S6. Percentage of desired reads... ...targeting *ALS*.. 12
Supplementary Table S7. Percentage of desired reads... ...targeting *ABI1*... 12
Supplementary Table S8. Percentage of desired reads... ...targeting *GL1*.. 13
Supplementary Table S9. Percentage of desired reads... ...targeting *EPSPS* and *ALS*........................... 13
Supplementary Table S10. Percentage of desired reads... ...targeting *ABI1* and *GL1*............................ 13
Supplementary Table S11. Inheritance of homozygous mutations in prime-edited T1 lines..................... 14
Supplementary Table S12. Mutation efficiencies... ...redesigned rtT for *ALS*-W574M mutation............... 15
Supplementary Methods 16
Supplementary Sequence S1. 492 (Csy4-PEmax)... 18
Supplementary Sequence S2. 496c (Csy4-PE6c).. 20
Supplementary Sequence S3. 496d (Csy4-PE6d) 21
Supplementary Sequence S4. 4A92 (Csy4-P2A-PEmax).. 22
Supplementary Sequence S5. 4A96c (Csy4-P2A-PE6c) 23
Supplementary Sequence S6. 4A96d (Csy4-P2A-PE6d)... 24
Supplementary Sequence S7. RNA cassette V1... 25
Supplementary Sequence S8. RNA cassette V2... 26
Supplementary Sequence S9. RNA cassette V1×2... 27

Supplementary Figure S1. Annotated genomic DNA sequences... ...and nicking sgRNAs

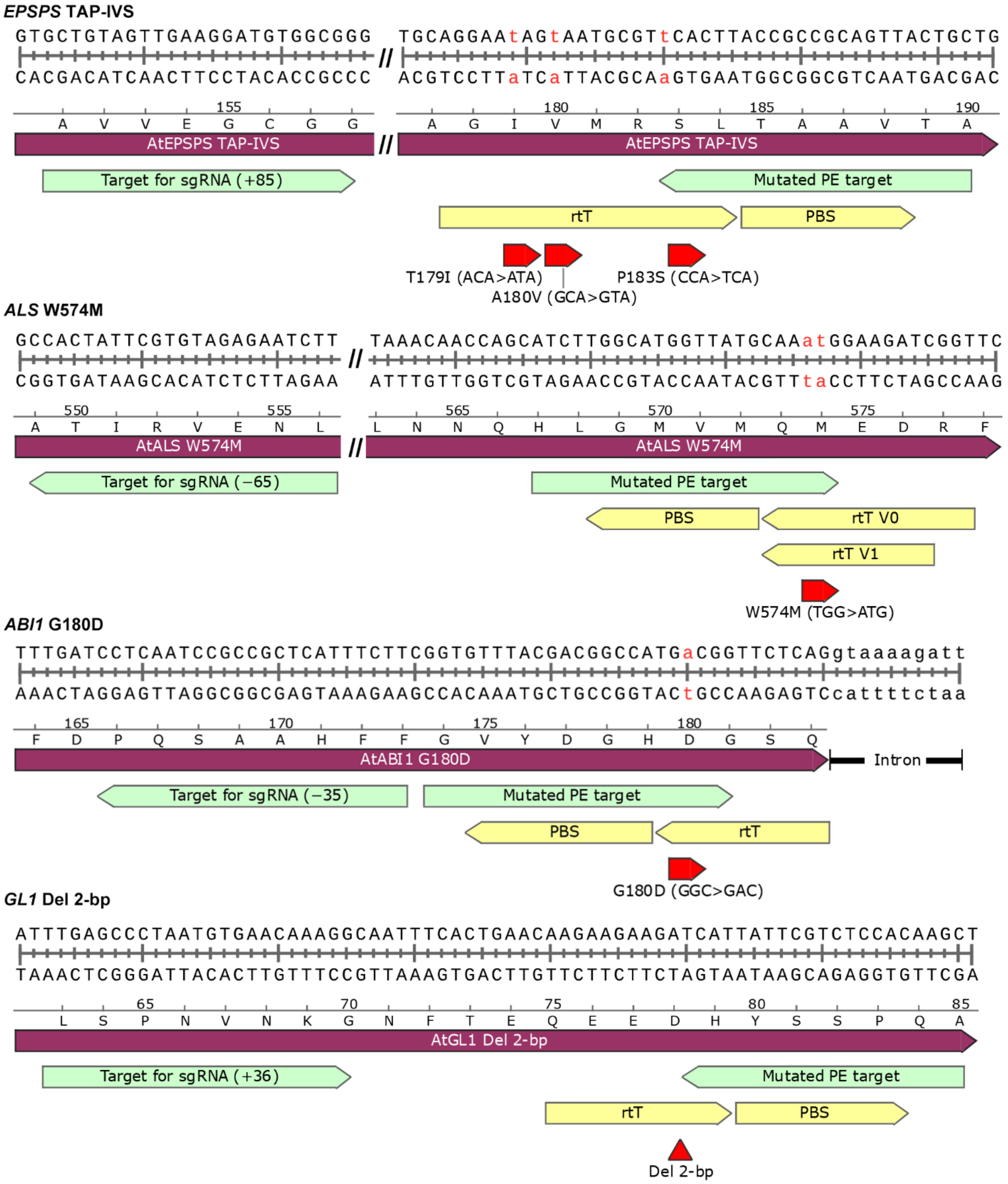

Supplementary Figure S1. Annotated genomic DNA sequences related to targeted mutations, pegRNAs, and nicking sgRNAs. Mutated nucleotides are indicated in lowercase and red. The first nucleotide following the pegRNA-induced nick is defined as position +1, and the positions of sgRNA-induced nicks are numbered accordingly.

Supplementary Figure S2. Representative sequencing chromatograms ... ...Sc byproducts

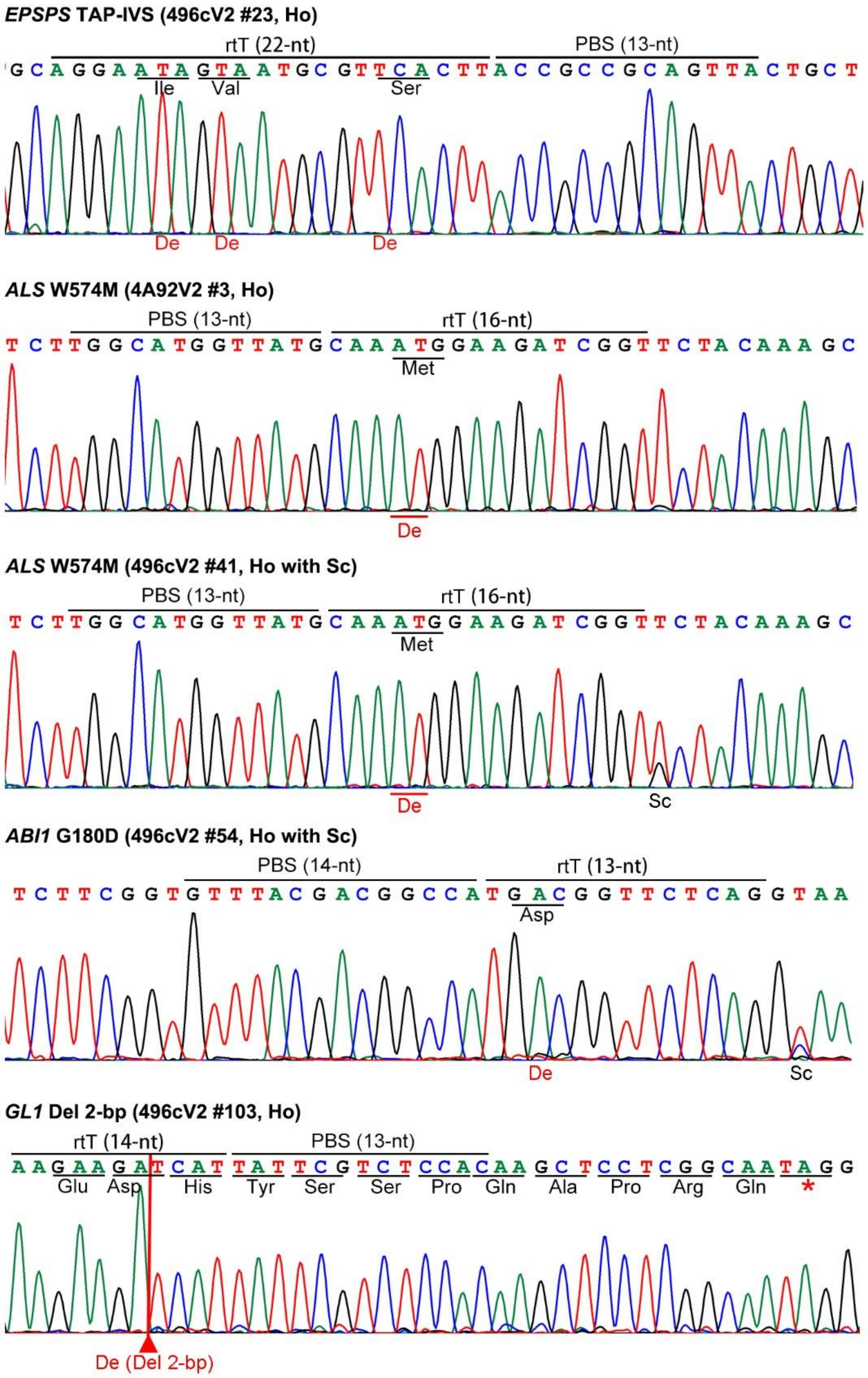

Supplementary Figure S2. Representative sequencing chromatograms indicative of the homozygous mutations in prime-edited lines with or without Sc byproducts. De, desired edit(s). Sc, pegRNA scaffold-derived byproducts.

Supplementary Figure S3. Reads-based editing efficiencies… ...across four targets

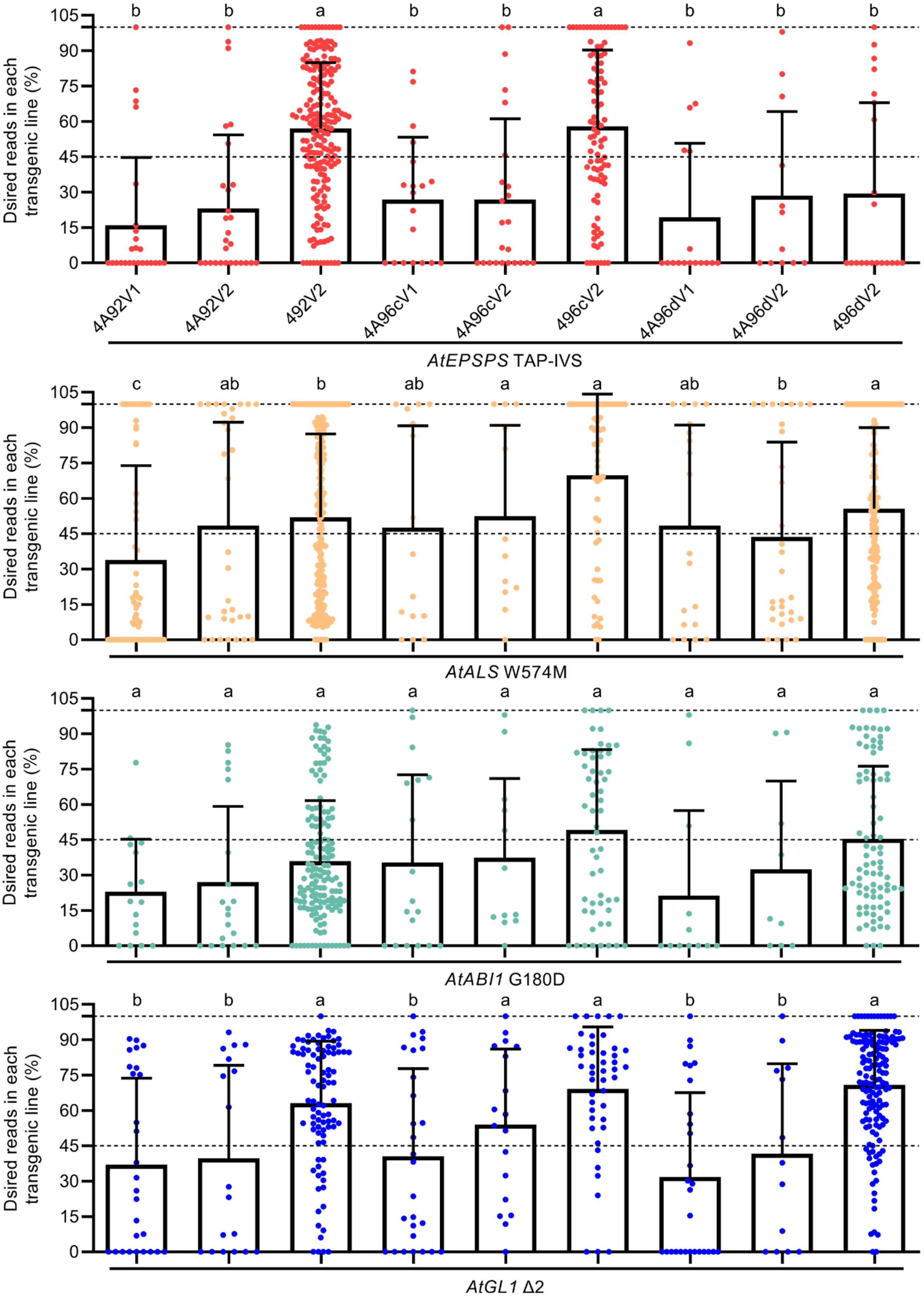

Supplementary Figure S3. Reads-based editing efficiencies of the six types of PEs in combination with the two RNA cassettes across four targets. Each dot represents the percentage of desired reads from an independent T1 transgenic line. “Desired” reads include those harboring desired edits and the pegRNA scaffold-derived byproducts to more precisely quantify prime-editing efficacy. Columns and error bars represent the mean and SD, respectively. One-way ANOVA followed by Tukey’s multiple-comparisons test was used; bars sharing a lowercase letter indicate no significant difference ($P > 0.05$).

Supplementary Figure S4. Phenotypes of T2 plants from T1 homozygous mutants.

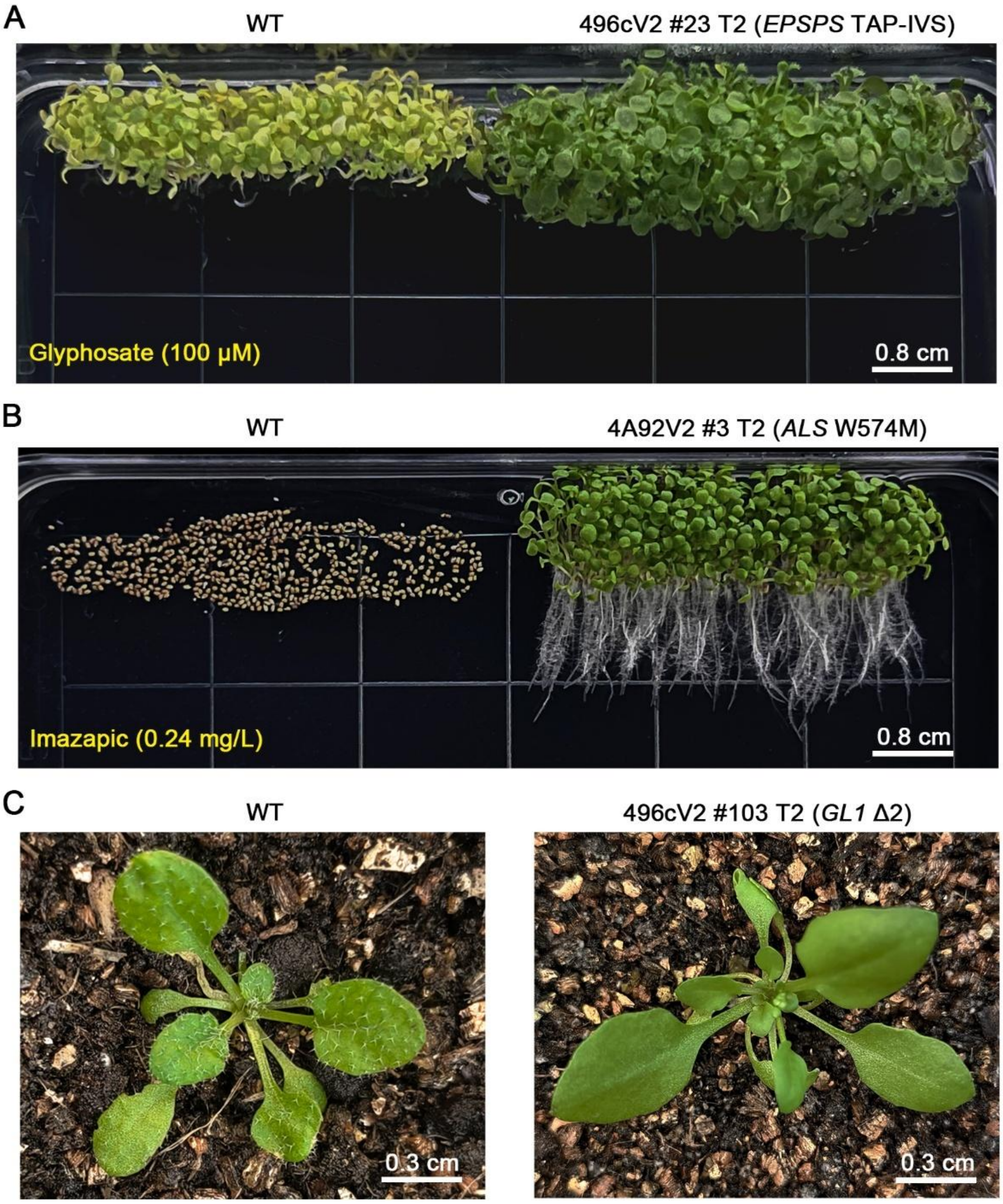

Supplementary Figure S4. Phenotypes of T2 plants from T1 homozygous mutants. Wild-type *Columbia*-0 and T2 seeds from T1 prime-edited lines with homozygous *EPSPS* TAP-IVS (A) or *ALS* W574M (B) mutations were sown on MS medium supplemented with the corresponding herbicides, vernalized at 4 °C for 3 d, and then grown under long-day conditions (16 h light/8 h dark) at 22 °C for 7 d before photographs were taken. For examination of the glabrous phenotypes of T2 plants from a T1 line with the homozygous *GL1* Δ2 mutation (C), wild-type Columbia-0 seeds and T2 seeds (identified as T-DNA-free via counter-selection using red fluorescence under a fluorescent stereomicroscope) were sown on MS medium, vernalized at 4 °C for 3 d, and then grown under long-day conditions (16 h light/8 h dark) at 22 °C for 9 d. The seedlings were then transplanted to soil and grown for 8 d before photographs were taken.

Supplementary Figure S5. Redesign of rtT of pegRNA... ...eliminated the Sc byproducts

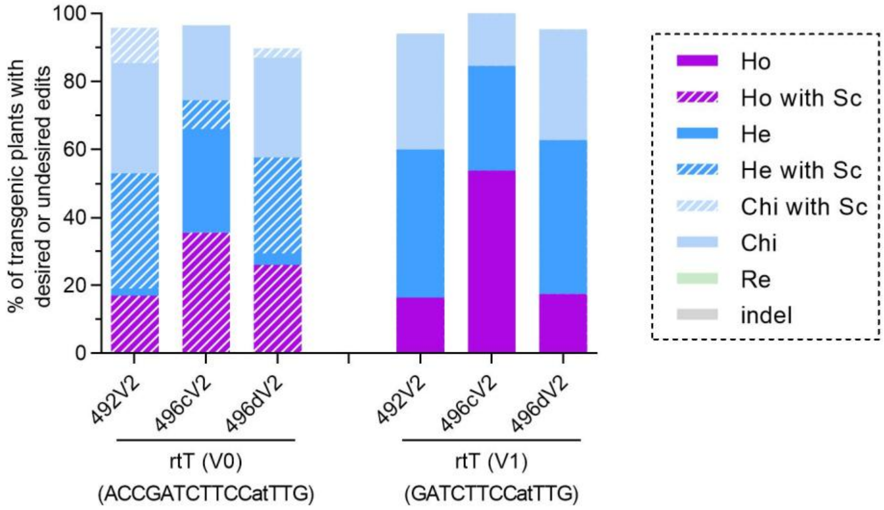

Supplementary Figure S5. Redesign of rtT of pegRNA for the *ALS*-W574M mutation eliminated the Sc byproducts. Ho, He, and Chi are homozygous, heterozygous, and chimeric mutant lines, respectively. Re, DNA repair-derived byproducts with only some of the target bases edited when installing multiple-base substitution edits. Sc, pegRNA scaffold-derived byproducts. Ho or He with Sc, homozygous or heterozygous mutant lines harboring desired edits along with the pegRNA scaffold-derived mutations. The original (V0) and Redesigned (V1) rtT sequences are indicated. Data for V0 is consistent with that shown in Fig. 1D.

Supplementary Table S1. Sequences related to pegRNAs and nicking sgRNAs

Supplementary Table S1. Sequences related to pegRNAs and nicking sgRNAs

Target	Gene ID	mutations	Name	Sequence
EPSPS	At1G48860	TAP-IVS	Target for pegRNA	GCAGTAACTGCGGCGGTAAGTGG
			rtT/PBS/Linker	AGGAAtAGtAATGCGTtCACTT /ACCGCCGCAG/TTAAGATATAT
			Target for nicking sgRNA	GCTGTAGTTGAAGGATGTGGCGG
ALS	At3G48560	W574M	Target for pegRNA	ATCTTGGCATGGTTATGCAATGG
			rtT-V0/PBS/Linker	ACCGATCTTCCatTTG/CATAACCATGCCA /CTCTACCT
			rtT-V1/PBS/Linker	GATCTTCCatTTG/CATAACCATGCCA /CTCTACC
			Target for nicking sgRNA	AAGATTCTCTACACGAATAGTGG
ABI1	At4G26080	G180D	Target for pegRNA	GGTGTTTACGACGGCCATGGCGG
			rtT/PBS/Linker	CTGAGAACCGtCA/TGGCCGTCGTAAAC /TAATTAAA
			Target for nicking sgRNA	AAGAAATGAGCGGCGGATTGAGG
GL1	At3G27920	The 2-bp deletion	Target for pegRNA	GCTTGTGGAGACGAATAATGAGG
			rtT/PBS/Linker	AAGAAGAAGA^TCAT/TATTCGTCTCCAC /TATATACG
			Target for nicking sgRNA	TTGAGCCCTAATGTGAACAAAGG

TAP-IVS, T179I, A180V and P183S.

Supplementary Table S2. Sequences of primers

Supplementary Table S2. Sequences of primers

Name	Sequence	Purpose
TAP-IDF	TCACAGTGAAAACAATCGTGCT	For Sanger sequencing
TAP-IDR	ACCGATTGAAAAGCATCAAAACA	
ALS-IDF	GTGTCGGGCAACATCAAATG	
ALS-IDR	TGCGGACAAATCACATCCAA	
ABI1-IDF	GAGATCAACGGCTCAGATGT	
ABI1-IDR	CCAAAGCCAAATGCATCCTC	
GL1-IDF	GAACCGCATCGTCAGAAA	
GL1-IDR	GAAATAACCCACCAACAC	
TAP-F	GGAGTGAGTACGGTGTGCTCACAGTGAAAACAATCGTGCT	For deep sequencing
TAP-R	GAGTTGGATGCTGGATGGACCGATTGAAAAGCATCAAAACA	
ALS-F	GGAGTGAGTACGGTGTGCGTGCAAGAGCTAGCCACTATTC	
ALS-R	GAGTTGGATGCTGGATGGGTTCGGGAATATCTCGTCCTC	
ABI1-F	GGAGTGAGTACGGTGTGCCCTTCAATCTTCTTCTGGT	
ABI1-R	GAGTTGGATGCTGGATGGTAGTTCGCTACCTACACAA	
GL1-F	GGAGTGAGTACGGTGTGCGTTGTAGACTGAGATGGATGAAT	
GL1-R	GAGTTGGATGCTGGATGGCGATTAAATAAAAAGACATGGTG	
Cas9-IDF	CGGCCTCGATATTGGGACTAACTCT	For transgene-free
Cas9-IDR	CTTATCTGTGGAGTCCACGAGCTTC	

Supplementary Table S3. Transformation efficiencies of 24… ...harboring uncleavable Csy4-PEs

Supplementary Table S3. Transformation efficiencies of 24 binary vectors harboring cleavable Csy4-P2A-PEs and 18 binary vectors harboring uncleavable Csy4-PEs

Target	PEs	Total number of T0 seeds	Number of T1 lines	Transformation efficiency (%)
EPSPS TAP-IVS	4A92V1	75100	25	0.0333
	4A92V2	75100	28	0.0373
	492V2	56433	417	0.7389
	4A96cV1	58621	19	0.0324
	4A96cV2	79775	24	0.0301
	496cV2	66100	192	0.2905
	4A96dV1	68400	17	0.0249
	4A96dV2	46517	20	0.0430
	496dV2	19950	64	0.3208
ALS W574M	4A92V1	153900	67	0.0435
	4A92V2	71838	32	0.0445
	492V2	67000	454	0.6776
	4A96cV1	48721	15	0.0308
	4A96cV2	43350	14	0.0323
	496cV2	72750	159	0.2186
	4A96dV1	50590	19	0.0376
	4A96dV2	58650	30	0.0512
	496dV2	74300	306	0.4118
ABI1 G180D	4A92V1	32300	16	0.0495
	4A92V2	39300	21	0.0534
	492V2	32650	149	0.4564
	4A96cV1	42300	18	0.0426
	4A96cV2	41600	12	0.0288
	496cV2	30700	56	0.1824
	4A96dV1	29700	12	0.0404
	4A96dV2	27350	10	0.0366
	496dV2	29900	85	0.2843
GL1 ∆2	4A92V1	65200	27	0.0414
	4A92V2	71550	18	0.0252
	492V2	55650	185	0.3324
	4A96cV1	61350	27	0.0440
	4A96cV2	72350	18	0.0249
	496cV2	81250	111	0.1366
	4A96dV1	64950	28	0.0431
	4A96dV2	53100	13	0.0245
	496dV2	57775	160	0.2769
EPSPS & *ALS*	492V1×2	59275	213	0.3593
	496cV1×2	41338	68	0.1645
	496dV1×2	50175	109	0.2172
ABI1 & *GL1*	492V1×2	36400	173	0.4753
	496cV1×2	34300	74	0.2157
	496dV1×2	57000	193	0.3386

Supplementary Table S4. Mutation efficiencies... ... in Arabidopsis

Supplementary Table S4. Mutation efficiencies of the six types of PEs in combination with the three RNA cassettes across the four targets in Arabidopsis

(See the XLSX spreadsheet)

Supplementary Table S5. Percentage of desired reads... ...targeting *EPSPS*

Supplementary Table S5. Percentage of desired reads in each line harboring one of nine PEs targeting *EPSPS*

(See the XLSX spreadsheet)

Supplementary Table S6. Percentage of desired reads... ...targeting *ALS*

Supplementary Table S6. Percentage of desired reads in each line edited by one of nine PEs targeting *ALS*

(See the XLSX spreadsheet)

Supplementary Table S7. Percentage of desired reads... ...targeting *ABI1*

Supplementary Table S7. Percentage of desired reads in each line edited by one of nine PEs targeting *ABI1*

(See the XLSX spreadsheet)

Supplementary Table S8. Percentage of desired reads... ...targeting *GL1*

Supplementary Table S8. Percentage of desired reads in each line edited by one of nine PEs targeting *GL1*

(See the XLSX spreadsheet)

Supplementary Table S9. Percentage of desired reads... ...targeting *EPSPS* and *ALS*

Supplementary Table S9. Percentage of desired reads in each line edited by one of three types of duplex PEs targeting *EPSPS* and *ALS*

(See the XLSX spreadsheet)

Supplementary Table S10. Percentage of desired reads... ...targeting *ABI1* and *GL1*

Supplementary Table S10. Percentage of desired reads in each line edited by one of three types of duplex PEs targeting *ABI1* and *GL1*

(See the XLSX spreadsheet)

Supplementary Table S11. Inheritance of homozygous mutations in prime-edited T1 lines

Supplementary Table S11. Inheritance of homozygous mutations in prime-edited T1 lines

Mutation	T1 homozygous mutant line	Ratio of T2 plants identified as T-DNA-free	Ratio of T-DNA-free T2 plants identified as homozygous mutants
EPSPS TAP-IVS	496cV2-#23	26.44% (23/87)	100%
	496cV2-#35	38.10% (32/84)	100%
	496dV2-#95	31.25% (15/48)	100%
ALS W574M	4A92V2-#3	27.08% (13/48)	100%
	4A96cV1-#57	25.53% (12/47)	100%
GL1 Δ2	496cV2-#58	33.33% (15/45)	100%
	496cV2-#84	32.61% (15/46)	100%
	496cV2-#85	37.78% (17/45)	100%

T-DNA-free T2 plants were obtained by PCR counter-selection. The mutations in the T-DNA-free T2 plants were analyzed by deep sequencing of PCR amplicons with 5% threshold. Lines with 100% deep-sequencing reads representing only one type of mutation with desired edits were scored as homozygous.

Supplementary Table S12. Mutation efficiencies... ...redesigned rtT for *ALS*-W574M mutation

Supplementary Table S12. Mutation efficiencies of three types of PEs with the redesigned rtT for *ALS*-W574M mutation

Target	PEs	Ratio of edited lines				
		Ho (%)	He (%)	Chi (%)	Sc (%)	Indel (%)
ALS W574M	492V2	16.47 (14/85)	43.53 (37/85)	34.12 (29/85)	0.00 (0/85)	0.00 (0/85)
	496cV2	53.85 (7/13)	30.77 (4/13)	15.38 (2/13)	0.00 (0/13)	0.00 (0/13)
	496dV2	17.44 (15/86)	45.35 (39/86)	32.56 (28/86)	0.00 (0/86)	0.00 (0/86)

The transgenic plants were analyzed by deep sequencing of PCR amplicons with 5% threshold. Lines with 100% deep-sequencing reads representing only one type of mutation with desired edits were scored as homozygous. Non-homozygous lines containing more than 45% deep sequencing reads representing desired edits were scored as heterozygous. Non-homozygous and non-heterozygous mutant lines were scored as Chi, Sc, or Indel lines when the main mutation types were desired edits, Sc byproducts (byproducts derived from the pegRNA scaffold), or Indel byproducts, respectively. Mutation efficiency was calculated as the proportion of mutant plants relative to the total number of transgenic plants.

Supplementary Methods

The sequences related to epegRNAs and nicking sgRNAs are listed in Supplementary Table 11, and all primers used in this study are listed in Supplementary Table 12. The coding sequences of PE proteins, with or without the P2A linker, are presented in Supplementary sequences 1–6, and the sequences of RNA cassettes V1, V2, and V1×2 are provided in Supplementary sequences 7–9.

Vector construction

The *Sbf*I-*Sac*I fragments of pG3H-PE6c and pG3H-PE6d (Cao et al. 2024) were replaced with the *Sbf*I-*Sac*I fragment from pG3H-4A92 (Lu et al. 2026), yielding pG3H-4A96c and pG3H-4A96d. Similarly, the *Sbf*I-*Sac*I fragments of pG3H-PE6c and pG3H-PE6d (Cao et al. 2024) were replaced with the corresponding *Sbf*I-*Sac*I fragment of pG3H-492 (Lu et al. 2026), resulting in generation of pG3H-496c and pG3H-496d. Subsequently, the *Xba*I-*Sac*II fragment within the T-DNA region of pBG-Lb12-RRVL (Xin et al. 2025)was replaced with the corresponding *Xba*I-*Sac*II fragments of pG3H-4A9* or pG3H-49* (* = 2, 6c, or 6d), resulting in generation of pH-4A92.0, pH-492.0, pH-4A96c.0, pH-496c.0, pH-4A96d.0, and pH-496d.0. The *Hind*III-*Spe*I fragment of pG3H-4A92 was then used to replace the corresponding fragments of pH-4A9*.0 or pH-49*.0 (* = 2, 6c, or 6d), resulting in pH-4A92, pH-492, pH-4A96c, pH-496c, pH-4A96d, and pH-496d. The synthetic fragments harboring 8 RNA cassettes V1 or V2 were digested with *Bsa*I and ligated with 3 *Bsa*I-digested pH-4A9* (* = 2, 6c, or 6d), producing 24 PEs with RNA cassettes V1 or V2 targeting *EPSPS*, *ALS*, *ABI1*, or *GL1*. The synthetic fragments harboring 4 RNA cassettes V2 were digested with *Bsa*I and ligated with 3 *Bsa*I-digested pH-49* (* = 2, 6c, or 6d), producing 12 PEs with RNA cassette V2 targeting *EPSPS*, *ALS*, *ABI1*, or *GL1*. The synthetic fragments harboring 2 RNA cassettes V1×2 were digested with *Bsa*I and ligated with 3 *Bsa*I-digested pH-49* (* = 2, 6c, or 6d), producing 6 PEs with RNA cassette V1×2 targeting *EPSPS*/*ALS* or *ABI1/GL1*. The synthetic fragment harboring one RNA cassette V2 with improved rtT (V1) for *ALS* W574 mutation was digested with *Bsa*I and ligated with 3 *Bsa*I-digested pH-49* (* = 2, 6c, or 6d), producing 3 PEs with RNA cassette V2 targeting *ALS*.

Generation of transgenic plants and analysis of transformation and prime-editing efficiencies

The 30 PE vectors were introduced into *Agrobacterium tumefaciens* strain GV3101. Transgenic plants were generated in the *Arabidopsis thaliana* accession Col-0 via the floral dip method. T0 seeds were harvested and weighed, and seed number was calculated according to the average 1,000-seed weight (20 mg/1,000 seeds).

T1 transformants were selected on Murashige and Skoog (MS) medium supplemented with 25 mg/L Hygromycin B. T1 seedlings or leaf tissues were subjected to genomic DNA extraction. To analyze prime-editing–induced mutations, genomic regions encompassing the target sites were PCR-amplified using primers listed in Supplementary Table 12 for Sanger or deep sequencing. The depth of deep sequencing is about 10 k reads for each PCR amplicon. The transgenic plants were analyzed by deep sequencing of PCR amplicons with 5% threshold (Sun et al. 2024). Lines with 100% deep-sequencing reads representing only one type of mutation with desired edits were scored as homozygous. Non-homozygous lines containing more than 45% deep sequencing reads representing desired edits were scored as heterozygous. Non-homozygous and non-heterozygous mutant lines were scored as Chi, Re, Sc, or Indel lines when the main mutation types were desired edits, Re byproducts (undesired edits derived from unbiased repair of heteroduplex DNA with multiple edits), Sc byproducts (byproducts derived from the pegRNA scaffold), or Indel byproducts, respectively. Mutation efficiency was calculated as the proportion of mutant plants relative to the total number of transgenic plants.

Supplementary References

Cao Z, Sun W, Qiao D, Wang J, Li S, Liu X, Xin C, Lu Y, Gul SL, Wang XC, Chen QJ (2024) PE6c greatly enhances prime editing in transgenic rice plants. J Integr Plant Biol **66:** 1864-1870

Lu Y, Qiao D, Wang J, Sun W, Cao Z, Lu M, Chai Y, Jiang Y, Xin C, Liu X, Li S, Gul SL, Chen QJ (2026) Two Highly Efficient Prime Editing Systems Based on the Csy4 CRISPR Endonuclease. Plant Biotechnol J **24:** 828-830

Sun T, Liu Q, Chen X, Hu F, Wang K (2024) Hi-TOM 2.0: an improved platform for high-throughput mutation detection. Sci China Life Sci **67:** 1532-1534

Xin C, Lu Y, Gul SL, Sun W, Cao Z, Kong X, Fan K, Li S, Liu X, Wang XC, Chen QJ (2025) Never-germinating Arabidopsis seeds with LbCas12a-induced mutations in 6 clade A type 2C protein phosphatase genes. Plant Physiol **198**

Supplementary Sequence S1. 492 (Csy4-PEmax)

Csy4-2xSGGS-NLS-2xSGGS-SpCas9KK-H840A-2xSGGS-NLS-2xSGGS-PEmaxRT-SGGS- NLS-NLS$^{c\text{-}Myc}$

ATGGACCACTACCTGGACATCAGGCTCAGGCCGGACCCTGAGTTCCCGCCTGCCCAGCTGATGTCCGTGCTGTTCGG
CAAGCTGCATCAGGCTCTCGTTGCTCAGGGCGGCGATCGCATCGGCGTCTCATTCCCTGATCTGGACGAGTCTAGGA
GCAGGCTGGGCGAGAGGCTCCGCATCCATGCCAGCGCTGACGACCTGAGGGCTCTGCTCGCCAGGCCGTGGCTGG
AGGGCCTCAGGGACCATCTCCAGTTCGGCGAGCCTGCTGTTGTGCCACATCCAACACCGTACAGGCAGGTCAGCAG
GGTGCAGGCCAAGTCCAATCCTGAGAGGCTGCGCAGGCGCCTCATGAGGCGCCATGATCTGTCTGAGGAGGAGGCT
AGGAAGCGCATCCCTGATACCGTTGCTAGGGCGCTGGATCTGCCATTCGTCACACTGCGCTCACAGAGCACTGGCCA
GCACTTCAGGCTGTTCATCAGGCATGGCCCACTCCAGGTCACTGCTGAGGAGGGCGGCTTCACATGCTACGGCCTCT
CCAAGGGCGGCTTCGTTCCGTGGTTCTCAGGCGGCTCATCTGGCGGGTCAAAGCGCACAGCCGACGGCTCTGAGTT
CGAGAGCCCTAAGAAGAAGCGCAAGGTGTCAGGCGGCTCTTCAGGCGGCAGCGACAAGAAGTACTCGATCGGCCTC
GATATTGGGACTAACTCTGTTGGCTGGGCCGTGATCACCGACGAGTACAAGGTGCCCTCAAAGAAGTTCAAGGTCCT
GGGCAACACCGATCGGCATTCCATCAAGAAGAATCTCATTGGCGCTCTCCTGTTCGACAGCGGCGAGACGGCTGAG
GCTACGCGGCTCAAGCGCACCGCCCGCAGGCGGTACACGCGCAGGAAGAATCGCATCTGCTACCTGCAGGAGATTT
TCTCCAACGAGATGGCGAAGGTTGACGATTCTTTCTTCCACAGGCTGGAGGAGTCATTCCTCGTGGAGGAGGATAAG
AAGCACGAGCGGCATCCAATCTTCGGCAACATTGTCGACGAGGTTGCCTACCACGAGAAGTACCCTACGATCTACCA
TCTGCGGAAGAAGCTCGTGGACTCCACAGATAAGGCGGACCTCCGCCTGATCTACCTCGCTCTGGCCCACATGATTA
AGTTCAGGGGCCATTTCCTGATCGAGGGGGATCTCAACCCGGACAATAGCGATGTTGACAAGCTGTTCATCCAGCTC
GTGCAGACGTACAACCAGCTCTTCGAGGAGAACCCCATTAATGCGTCAGGCGTCGACGCGAAGGCTATCCTGTCCG
CTAGGCTCTCGAAGTCTCGGAAGCTCGAGAACCTGATCGCCCAGCTGCCGGGCGAGAAGAAGAACGGCCTGTTCGG
GAATCTCATTGCGCTCAGCCTGGGGCTCACGCCCAACTTCAAGTCGAATTTCGATCTCGCTGAGGACGCCAAGCTGC
AGCTCTCCAAGGACACATACGACGATGACCTGGATAACCTCCTGGCCCAGATCGGCGATCAGTACGCGGACCTGTTC
CTCGCTGCCAAGAATCTGTCGGACGCCATCCTCCTGTCTGATATTCTCAGGGTGAACACCGAGATTACGAAGGCTCC
GCTCTCAGCCTCCATGATCAAGCGCTACGACGAGCACCATCAGGATCTGACCCTCCTGAAGGCGCTGGTCAGGCAG
CAGCTCCCCGAGAAGTACAAGGAGATCTTCTTCGATCAGTCGAAGAACGGCTACGCTGGGTACATTGACGGCGGG
CCTCTCAGGAGGAGTTCTACAAGTTCATCAAGCCGATTCTGGAGAAGATGGACGGCACGGAGGAGCTGCTGGTGAA
GCTCAAGCGCGAGGACCTCCTGAGGAAGCAGCGGACATTCGATAACGGCAGCATCCCACACCAGATTCATCTCGGG
GAGCTGCACGCTATCCTGAGGAGGCAGGAGGACTTCTACCCTTTCCTCAAGGATAACCGCGAGAAGATCGAGAAGAT
TCTGACTTTCAGGATCCCGTACTACGTCGGCCCACTCGCTAGGGGCAACTCCCGCTTCGCTTGGATGACCCGCAAGT
CAGAGGAGACGATCACGCCGTGGAACTTCGAGGAGGTGGTCGACAAGGGCGCTAGCGCTCAGTCGTTCATCGAGAG
GATGACGAATTTCGACAAGAACCTGCCAAATGAGAAGGTGCTCCCTAAGCACTCGCTCCTGTACGAGTACTTCACAGT
CTACAACGAGCTGACTAAGGTGAAGTATGTGACCGAGGGCATGAGGAAGCCGGCTTTCCTGTCTGGGGAGCAGAAG
AAGGCCATCGTGGACCTCCTGTTCAAGACCAACCGGAAGGTCACGGTTAAGCAGCTCAAGGAGGACTACTTCAAGAA
GATTGAGTGCTTCGATTCGGTCGAGATCTCTGGCGTTGAGGACCGCTTCAACGCCTCCCTGGGGACCTACCACGATC
TCCTGAAGATCATTAAGGATAAGGACTTCCTGGACAACGAGGAGAATGAGGATATCCTCGAGGACATTGTGCTGACA
CTCACTCTGTTCGAGGACCGGGAGATGATCGAGGAGCGCCTGAAGACTTACGCCCATCTCTTCGATGACAAGGTCAT
GAAGCAGCTCAAGAGGAGGAGGTACACCGGCTGGGGGAGGCTGAGCAGGAAGCTCATCAACGGCATTCGGGACAA
GCAGTCCGGGAAGACGATCCTCGACTTCCTGAAGAGCGATGGCTTCGCGAACCGCAATTTCATGCAGCTGATTCACG
ATGACAGCCTCACATTCAAGGAGGATATCCAGAAGGCTCAGGTGAGCGGCCAGGGGGACTCGCTGCACGAGCATAT
CGCGAACCTCGCTGGCTCGCCAGCTATCAAGAAGGGGATTCTGCAGACCGTGAAGGTTGTGGACGAGCTGGTGAAG
GTCATGGGCAGGCACAAGCCTGAGAACATCGTCATTGAGATGGCCCGGGAGAATCAGACCACGCAGAAGGGCCAGA
AGAACTCACGCGAGAGGATGAAGAGGATCGAGGAGGGCATTAAGGAGCTGGGGTCCCAGATCCTCAAGGAGCACCC
GGTGGAGAACACGCAGCTGCAGAATGAGAAGCTCTACCTGTACTACCTCCAGAATGGCCGCGATATGTATGTGGACC
AGGAGCTGGATATTAACAGGCTCAGCGATTACGACGTCGATGCCATCGTTCCACAGTCATTCCTGAAGGATGACTCC
ATTGACAACAAGGTCCTCACCAGGTCGGACAAGAACCGGGGCAAGTCTGATAATGTTCCTTCAGAGGAGGTCGTTAA
GAAGATGAAGAACTACTGGCGCCAGCTCCTGAATGCCAAGCTGATCACGCAGCGGAAGTTCGATAACCTCACAAAGG
CTGAGAGGGGCGGGCTCTCTGAGCTGGACAAGGCGGGCTTCATCAAGAGGCAGCTGGTCGAGACACGGCAGATCA
CTAAGCACGTTGCGCAGATTCTCGACTCACGGATGAACACTAAGTACGATGAGAATGACAAGCTGATCCGCGAGGTG
AAGGTCATCACCCTGAAGTCAAAGCTCGTCTCCGACTTCAGGAAGGATTTCCAGTTCTACAAGGTTCGGGAGATCAAC
AATTACCACCATGCCCATGACGCGTACCTGAACGCGGTGGTCGGCACAGCTCTGATCAAGAAGTACCCAAAGCTCGA
GAGCGAGTTCGTGTACGGGGACTACAAGGTTTACGATGTGAGGAAGATGATCGCCAAGTCGGAGCAGGAGATTGGC
AAGGCTACCGCCAAGTACTTCTTCTACTCTAACATTATGAATTTCTTCAAGACAGAGATCACTCTGGCCAATGGCGAGA
TCCGGAAGCGCCCCCTCATCGAGACGAACGGCGAGACGGGGGAGATCGTGTGGGACAAGGGCAGGGATTTCGCGA
CCGTCAGGAAGGTTCTCTCCATGCCACAAGTGAATATCGTCAAGAAGACAGAGGTCCAGACTGGCGGGTTCTCTAAG

GAGTCAATTCTGCCTAAGCGGAACAGCGACAAGCTCATCGCCCGCAAGAAGGACTGGGATCCGAAGAAGTACGGCG
GGTTCGACAGCCCCACTGTGGCCTACTCGGTCCTGGTTGTGGCGAAGGTTGAGAAGGGCAAGTCCAAGAAGCTCAA
GAGCGTGAAGGAGCTGCTGGGGATCACGATTATGGAGCGCTCCAGCTTCGAGAAGAACCCGATCGATTTCCTGGAG
GCGAAGGGCTACAAGGAGGTGAAGAAGGACCTGATCATTAAGCTCCCCAAGTACTCACTCTTCGAGCTGGAGAACGG
CAGGAAGCGGATGCTGGCTTCCGCTGGCGAGCTGCAGAAGGGGAACGAGCTGGCTCTGCCGTCCAAGTATGTGAAC
TTCCTCTACCTGGCCTCCCACTACGAGAAGCTCAAGGGCAGCCCCGAGGACAACGAGCAGAAGCAGCTGTTCGTCG
AGCAGCACAAGCATTACCTCGACGAGATCATTGAGCAGATTTCCGAGTTCTCCAAGCGCGTGATCCTGGCCGACGCG
AATCTGGATAAGGTCCTCTCCGCGTACAACAAGCACCGCGACAAGCCAATCAGGGAGCAGGCTGAGAATATCATTCA
TCTCTTCACCCTGACGAACCTCGGCGCCCCTGCTGCTTTCAAGTACTTCGACACAACTATCGATCGCAAGAGGTACAC
AAGCACTAAGGAGGTCCTGGACGCGACCCTCATCCACCAGTCGATTACCGGCCTCTACGAGACGCGCATCGACCTG
TCTCAGCTCGGGGGCGACTCAGGCGGCTCATCTGGCGGGTCAAAGCGCACAGCCGACGGCTCTGAGTTCGAGAGC
CCTAAGAAGAAGCGCAAGGTGTCAGGCGGCTCTTCAGGCGGCAGCACCCTGAACATTGAGGACGAGTACCGGCTGC
ACGAGACGAGCAAGGAGCCAGACGTTTCGCTCGGCAGCACTTGGCTCTCTGACTTCCCACAGGCTTGGGCCGAGAC
TGGCGGCATGGGCCTGGCCGTGCGCCAGGCTCCACTGATCATCCCTCTGAAGGCGACCTCCACCCCGGTTTCTATT
AAGCAGTACCCGATGAGCCAGGAGGCCAGGCTGGGGATCAAGCCACACATTCAGCGGCTGCTGGACCAGGGCATC
CTGGTGCCATGCCAGTCCCCGTGGAATACTCCGCTCCTGCCGGTGAAGAAGCCTGGGACAAACGACTACAGGCCGG
TTCAGGATCTCAGGGAGGTGAACAAGCGCGTGGAGGACATCCATCCGACAGTGCCGAACCCGTACAATCTGCTGTC
GGGCCTGCCTCCGAGCCACCAGTGGTACACCGTCCTGGACCTCAAGGACGCTTTCTTCTGCCTGCGGCTGCACCCG
ACGTCTCAGCCGCTGTTCGCGTTCGAGTGGCGCGACCCAGAGATGGGCATTTCCGGCCAGCTGACCTGGACACGCC
TACCCCAGGGCTTCAAGAACTCCCCGACTCTCTTCAACGAGGCTCTCCACCGGGATCTCGCGGACTTCAGGATTCAG
CATCCCGATCTGATCCTGCTCCAGTATGTTGACGACCTCCTCCTGGCCGCGACGTCGGAGCTGGACTGCCAGCAGG
GCACCCGGGCGCTGCTGCAGACACTGGGCAATCTGGGGTACCGCGCCTCTGCGAAGAAGGCGCAGATCTGCCAGA
AGCAAGTGAAGTACCTGGGCTACCTCCTGAAGGAGGGCCAGCGCTGGCTCACTGAGGCGAGGAAGGAGACTGTTAT
GGGCCAGCCCACTCCAAAGACTCCGAGGCAGCTCAGGGAGTTCCTCGGCAAGGCTGGGTTCTGCCGCCTGTTCATC
CCTGGGTTCGCTGAGATGGCTGCGCCGCTCTACCCGCTGACTAAGCCGGGGACACTGTTCAACTGGGGGCCAGACC
AGCAGAAGGCGTACCAGGAGATTAAGCAGGCGCTGCTGACGGCCCCAGCGCTCGGCCTACCAGACCTGACGAAGC
CGTTCGAGCTGTTCGTTGACGAGAAGCAGGGGTACGCGAAGGGCGTGCTGACACAGAAGCTGGGGCCTTGGCGCC
GCCCGGTCGCGTACCTGTCGAAGAAGCTGGACCCAGTCGCTGCTGGGTGGCCTCCATGCCTCCGGATGGTCGCTGC
TATTGCGGTTCTGACCAAGGATGCGGGGAAGCTCACAATGGGGCAGCCTCTCGTGATCCTGGCTCCACATGCGGTG
GAGGCGCTGGTGAAGCAGCCACCGGACCGGTGGCTGTCGAACGCTCGGATGACACACTACCAGGCGCTCCTCCTC
GATACAGACCGGGTTCAGTTCGGGCCTGTGGTTGCTCTGAACCCAGCCACACTGCTGCCACTCCCTGAGGAGGGCC
TCCAGCACAATTGCCTCGACATCCTGGCTGAGGCGCACGGCACCCGCCCTGATCTCACCGACCAGCCTCTGCCAGA
TGCTGACCACACCTGGTACACGGATGGGTCCTCGCTGCTGCAGGAGGGCCAGAGGAAGGCGGGCGCCGCCGTCAC
CACAGAGACAGAGGTTATTTGGGCCAAGGCCCTACCGGCTGGCACCAGCGCCCAGCGCGCTGAGCTGATCGCGCT
GACTCAGGCGCTGAAGATGGCCGAGGGGAAGAAGCTCAATGTTTACACCGACTCGCGGTACGCGTTCGCTACAGCT
CACATTCATGGGGAGATCTACCGCCGGCGCGGGTGGCTGACTTCGGAGGGCAAGGAGATTAAGAATAAGGACGAGA
TCCTGGCCCTGCTCAAGGCGCTGTTCCTGCCGAAGCGCCTCTCAATCATTCACTGCCCGGGCCACCAGAAGGGCCA
TTCGGCCGAGGCTAGGGGCAATCGGATGGCTGACCAGGCGGCGCGGAAGGCGGCTATCACCGAGACTCCCGATAC
ATCTACCCTCCTGATCGAGAACTCGAGCCCATCTGGCGGCTCTAAGCGGACTGCGGATGGGTCTGAGTTCGAGTCAC
CAAAGAAGAAGAGGAAGGTGGGCTCTGGCCCTGCTGCTAAGCGCGTGAAGCTCGATTGA

The background colors of the nucleotide sequence correspond to the functional elements shown above.

Supplementary Sequence S2. 496c (Csy4-PE6c)

Csy4-2xSGGS-NLS-2xSGGS-SpCas9KK-H840A-2xSGGS-NLS-2xSGGS-PE6cRT-SGGS- NLS-NLS$^{c\text{-}Myc}$

ATGGACCACTACCTGGACATCAGGCTCAGGCCGGACCCTGAGTTCCCGCCTGCCCAGCTGATGTCCGTGCTGTTCGG
CAAGCTGCATCAGGCTCTCGTTGCTCAGGGCGGCGATCGCATCGGCGTCTCATTCCCTGATCTGGACGAGTCTAGGA
GCAGGCTGGGCGAGAGGCTCCGCATCCATGCCAGCGCTGACGACCTGAGGGCTCTGCTCGCCAGGCCGTGGCTGG
AGGGCCTCAGGGACCATCTCCAGTTCGGCGAGCCTGCTGTTGTGCCACATCCAACACCGTACAGGCAGGTCAGCAG
GGTGCAGGCCAAGTCCAATCCTGAGAGGCTGCGCAGGCGCCTCATGAGGCGCCATGATCTGTCTGAGGAGGAGGCT
AGGAAGCGCATCCCTGATACCGTTGCTAGGGCGCTGGATCTGCCATTCGTCACACTGCGCTCACAGAGCACTGGCCA
GCACTTCAGGCTGTTCATCAGGCATGGCCCACTCCAGGTCACTGCTGAGGAGGGCGGCTTCACATGCTACGGCCTCT
CCAAGGGCGGCTTCGTTCCGTGGTTCTCAGGCGGCTCATCTGGCGGGTCAAAGCGCACAGCCGACGGCTCTGAGTT
CGAGAGCCCTAAGAAGAAGCGCAAGGTGTCAGGCGGCTCTTCAGGCGGCAGCGAC//GACTCAGGCGGCTCATCTGG
CGGGTCAAAGCGCACAGCCGACGGCTCTGAGTTCGAGAGCCCTAAGAAGAAGCGCAAGGTGTCAGGCGGCTCTTCA
GGCGGCAGCATCTCAAGCTCTAAGCACACGCTGTCGCAGATGAACAAGGTGTCGAACATCGTCAAGGAGCCTGAGC
TGCCGGACATCTACAAGGAGTTCAAGGACATCACCGCCGATACTAACACGGAGAAGCTGCCCAAGCCAATCAAGGGC
CTGGAGTTCGAGGTTGAGCTGACCCAGGAGAACTACCGCCTGCCCATCAGGAACTACCCACTCACACCGGTGAAGAT
GCAGGCGATGAATGATGAGATCAACCAGGGCCTCAAGGGCGGCATCATCCGCGAGTCAAAGGCTATCAACGCCTGC
CCGGTCATCTTCGTTCCACGCAAGGAGGGCACCCTGAGGATGGTGGTTGACTACAGGCCACTGAACAAGTATGTCAA
GCCAAACGTGTACCCGCTCCCGCTGATTGAGCAGCTGCTCGCCAAGATCCAGGGCTCTACTATCTTCACGAAGCTCG
ATCTGAAGAGCGCTTACCACCAGATCCGGGTGCGCAAGGGCGATGAGCACAAGCTCGCCTTCCGCTGCCCACGCGG
CGTCTTCGAGTACCTCGTGATGCCGTACGGCATCAAGACAGCTCCAGCGCACTTCCAGTACTTCATCAACACGATCCT
GGGCGAGGCCAAGGAGTCGCATGTGGTGTGCTACATGGACGACATCCTGATTCATTCGAAGTCTGAGAGCGAGCAC
GTGAAGCACGTCAAGGATGTGCTGCAGAAGCTCAAGAATGCCAACCTCATCATCAATCAGGCGAAGTGCGAGTTCCA
CCAGTCTCAGGTCAAGTTCCTCGGCTACCACATCTCTGAGAAGGGCCTCACACCCTGCCAGGAGAACATCGACAAGG
TGCTCCAGTGGAAGCAGCCTAAGAATCAGAAGGAGCTGCGCCAGTTCCTCGGCCAGGTGAACTACCTCCGCAAGTTC
ATTCCAAAGACTTCACAGCTGACGCACCCACTGAACAAGCTGCTCAAGAAGGATGTGCGGTGGAAGTGGACTCCAAC
TCAGACGCAGGCTATCGAGAACATCAAGCAGTGCCTCGTGTCACCACCCGTGCTGCGCCACTTCGACTTCAGCAAGA
AGATCCTGCTGGAGACGGATGTGTCGGATGTCGCTGTCGGCGCTGTGCTCAGCCAGAAGCACGACGATGACAAGTA
CTACCCGGTTGGCTACTACTCAGCGAAGATGAGCAAGGCCCAGCTCAACTACTCTGTCTCAGACAAGGAGATGCTCG
CTATCATCAAGTCACTCGAGCACTGGCGCCACTACCTCGAGTCAACTATCGAGCCATTCAAGATCCTGACCGACCACA
GGAATCTGATCGGCCGCATCACTAACGAGTCGGAGCCAGAGAACAAGCGGCTCGCGAGGTGGCAGCTGTTCCTCCA
GGACTTCAACTTCGAGATCAACTACAGGCCAGGCTCGGCGAATCACATCGCCGACGCCCTGTCACGCATTGTTGATG
AGACAGAGCCGATTCCGAAGGACAACGAGGACAACAGCATCAACTTCGTCAACCAGATCAGCATCTCTGGCGGCTCT
AAGCGGACTGCGGATGGGTCTGAGTTCGAGTCACCAAAGAAGAAGAGGAAGGTGGGCTCTGGCCCTGCTGCTAAGC
GCGTGAAGCTCGATTGA

The background colors of the nucleotide sequence correspond to the functional elements shown above. “//” indicates that the sequence is omitted for concision (see the full sequence in 492)

Supplementary Sequence S3. 496d (Csy4-PE6d)

Csy4-2xSGGS-NLS-2xSGGS-SpCas9KK-H840A-2xSGGS-NLS-2xSGGS-PE6dRT-SGGS- NLS-NLS$^{c\text{-}Myc}$

ATGGACCACTACCTGGACATCAGGCTCAGGCCGGACCCTGAGTTCCCGCCTGCCCAGCTGATGTCCGTGCTGTTCGG
CAAGCTGCATCAGGCTCTCGTTGCTCAGGGCGGCGATCGCATCGGCGTCTCATTCCCTGATCTGGACGAGTCTAGGA
GCAGGCTGGGCGAGAGGCTCCGCATCCATGCCAGCGCTGACGACCTGAGGGCTCTGCTCGCCAGGCCGTGGCTGG
AGGGCCTCAGGGACCATCTCCAGTTCGGCGAGCCTGCTGTTGTGCCACATCCAACACCGTACAGGCAGGTCAGCAG
GGTGCAGGCCAAGTCCAATCCTGAGAGGCTGCGCAGGCGCCTCATGAGGCGCCATGATCTGTCTGAGGAGGAGGCT
AGGAAGCGCATCCCTGATACCGTTGCTAGGGCGCTGGATCTGCCATTCGTCACACTGCGCTCACAGAGCACTGGCCA
GCACTTCAGGCTGTTCATCAGGCATGGCCCACTCCAGGTCACTGCTGAGGAGGGCGGCTTCACATGCTACGGCCTCT
CCAAGGGCGGCTTCGTTCCGTGGTTCTCAGGCGGCTCATCTGGCGGGTCAAAGCGCACAGCCGACGGCTCTGAGTT
CGAGAGCCCTAAGAAGAAGCGCAAGGTGTCAGGCGGCTCTTCAGGCGGCAGCGAC//GACTCAGGCGGCTCATCTGG
CGGGTCAAAGCGCACAGCCGACGGCTCTGAGTTCGAGAGCCCTAAGAAGAAGCGCAAGGTGTCAGGCGGCTCTTCA
GGCGGCAGCACACTGAACATTGAGGATGAGTACAGGCTGCACGAGACTAGCAAGGAGCCAGACGTTAGCCTCGGCA
GCACATGGCTCTCTGACTTCCCACAGGCTTGGGCCGAGACTGGCGGCATGGGCCTGGCCGTGAGGCAGGCTCCACT
GATCATCCCTCTGAAGGCCACCAGCACACCTGTTAGCATCAAGCAGTACCCTATGTCACAGGAGGCCAGGCTCGGCA
TCAAGCCACACATTCAGCGGCTGCTGGACCAGGGCATCCTGGTGCCATGCCAGTCACCTTGGAACACACCACTCCTG
CCTGTCAAGAAGCCTGGGACTAACGACTACAGGCCCGTTCAGGATCTCAGGGAGGTCAACAAGCGCGTTGAGGACA
TCCATCCCAATGTGCCCAATCCGTACAATCTGCTGTCTGGCCTGCCTCCGTCACACCAGTGGTACACTGTCCTGGAC
CTCAAGGATGCGTTCTTCTGCCTGCGGCTGCACCCGACATCACAGCCGCTGTTCGCGTTCGAGTGGCGCGATCCAG
AGATGGGCATCAGCGGCCAGCTGACATGGACACGCCTGCCACAGGGCTTCAAGAACTCACCGACACTGTTCTGCGA
GGCTCTCCACAGGGATCTCGCTGACTTCAGGATTCAGCATCCCGATCTGATCCTGCTCCAGTACTACGATGACCTGC
TCCTGGCTGCTACATCTGAGCTGGACTGCCAGCAGGGCACACGCGCTCTGCTGCAGACACTGGGCAATCTCGGCTA
CAGGGCCAGCGCTAAGAAGGCGCAGATCTGCCAGAAGCAGGTCAAGTACCTGGGCTACCTGCTCAAGGAGGGCCA
GCGCTGGCTCACTGAGGCTCGCAAGGAGACTGTGATGGGCCAGCCTACACCTAAGACACCCAGGCAGCTCAGGGAG
TTCCTCGGCACTGCTGGGTTCTGCAGGCTGTGGATCCCTGGGTTCGCTGAGATGGCTGCGCCACTCTACCCACTGAC
TAAGACTGGCACACTGTTCAACTGGGGCCCAGATCAGCAGAAGGCGTACCAGGAGATCAAGCAGGCGCTGCTGACT
GCTCCTGCGCTCGGCCTGCCTGATCTGACCAAGCCATTCGAGCTGTTCGTTGATGAGAAGCAGGGCTACGCTAAGG
GCGTGCTGACACAGAAGCTCGGGCCTTGGCGCAGGCCAGTCGCGTACCTGTCCAAGAAGCTCGATCCAGTCGCTGC
TGGGTGGCCACCATGCCTCAGGATGGTCGCTGCTATCGCCGTTCTGACTAAGGATGCTGGCAAGCTCACTATGGGC
CAGCCACTCGTGATCCTGGCTCCACACGCCGTGGAGGCGCTGGTGAAGCAGCCACCTGATAGGTGGCTGTCTAACG
CTAGGATGACACACTACCAGGCGCTGCTGCTCGATACTGATAGGGTTCAGTTCGGGCCTGTGGTTGCTCTGAATCCA
GCCACACTGCTGCCACTCCCTGAGGAGGGCCTCCAGCACAACTGCCTCTCTGGCGGCTCTAAGCGGACTGCGGATG
GGTCTGAGTTCGAGTCACCAAAGAAGAAGAGGAAGGTGGGCTCTGGCCCTGCTGCTAAGCGCGTGAAGCTCGATTG
A

The background colors of the nucleotide sequence correspond to the functional elements shown above. “//” indicates that the sequence is omitted for concision (see the full sequence in 492)

Supplementary Sequence S4. 4A92 (Csy4-P2A-PEmax)

Csy4-P2A-NLS-SpCas9KK-H840A-2xSGGS-NLS-2xSGGS-PEmaxRT-SGGS- NLS-NLS$^{c\text{-}Myc}$

ATGGACCACTACCTGGACATCAGGCTCAGGCCGGACCCTGAGTTCCCGCCTGCCCAGCTGATGTCCGTGCTGTTCGG
CAAGCTGCATCAGGCTCTCGTTGCTCAGGGCGGCGATCGCATCGGCGTCTCATTCCCTGATCTGGACGAGTCTAGGA
GCAGGCTGGGCGAGAGGCTCCGCATCCATGCCAGCGCTGACGACCTGAGGGCTCTGCTCGCCAGGCCGTGGCTGG
AGGGCCTCAGGGACCATCTCCAGTTCGGCGAGCCTGCTGTTGTGCCACATCCAACACCGTACAGGCAGGTCAGCAG
GGTGCAGGCCAAGTCCAATCCTGAGAGGCTGCGCAGGCGCCTCATGAGGCGCCATGATCTGTCTGAGGAGGAGGCT
AGGAAGCGCATCCCTGATACCGTTGCTAGGGCGCTGGATCTGCCATTCGTCACACTGCGCTCACAGAGCACTGGCCA
GCACTTCAGGCTGTTCATCAGGCATGGCCCACTCCAGGTCACTGCTGAGGAGGGCGGCTTCACATGCTACGGCCTCT
CCAAGGGCGGCTTCGTTCCGTGGTTCGGCTCTGGCGCTACTAACTTCTCACTGCTGAAGCAGGCTGGCGATGTTGAG
GAGAATCCTGGCCCTATGAAGCGCACAGCCGATGGCAGCGAGTTCGAGTCACCTAAGAAGAAGCGCAAGGTGGAC//G
ACTCAGGCGGCTCATCTGGCGGGTCAAAGCGCACAGCCGACGGCTCTGAGTTCGAGAGCCCTAAGAAGAAGCGCAA
GGTGTCAGGCGGCTCTTCAGGCGGCAGCACCCTGAACATTGAGGACGAGTACCGGCTGCACGAGACGAGCAAGGA
GCCAGACGTTTCGCTCGGCAGCACTTGGCTCTCTGACTTCCCACAGGCTTGGGCCGAGACTGGCGGCATGGGCCTG
GCCGTGCGCCAGGCTCCACTGATCATCCCTCTGAAGGCGACCTCCACCCCGGTTTCTATTAAGCAGTACCCGATGAG
CCAGGAGGCCAGGCTGGGGATCAAGCCACACATTCAGCGGCTGCTGGACCAGGGCATCCTGGTGCCATGCCAGTC
CCCGTGGAATACTCCGCTCCTGCCGGTGAAGAAGCCTGGGACAAACGACTACAGGCCGGTTCAGGATCTCAGGGAG
GTGAACAAGCGCGTGGAGGACATCCATCCGACAGTGCCGAACCCGTACAATCTGCTGTCGGGCCTGCCTCCGAGCC
ACCAGTGGTACACCGTCCTGGACCTCAAGGACGCTTTCTTCTGCCTGCGGCTGCACCCGACGTCTCAGCCGCTGTTC
GCGTTCGAGTGGCGCGACCCAGAGATGGGCATTTCCGGCCAGCTGACCTGGACACGCCTACCCCAGGGCTTCAAGA
ACTCCCCGACTCTCTTCAACGAGGCTCTCCACCGGGATCTCGCGGACTTCAGGATTCAGCATCCCGATCTGATCCTG
CTCCAGTATGTTGACGACCTCCTCCTGGCCGCGACGTCGGAGCTGGACTGCCAGCAGGGCACCCGGGCGCTGCTG
CAGACACTGGGCAATCTGGGGTACCGCGCCTCTGCGAAGAAGGCGCAGATCTGCCAGAAGCAAGTGAAGTACCTGG
GCTACCTCCTGAAGGAGGGCCAGCGCTGGCTCACTGAGGCGAGGAAGGAGACTGTTATGGGCCAGCCCACTCCAAA
GACTCCGAGGCAGCTCAGGGAGTTCCTCGGCAAGGCTGGGTTCTGCCGCCTGTTCATCCCTGGGTTCGCTGAGATG
GCTGCGCCGCTCTACCCGCTGACTAAGCCGGGGACACTGTTCAACTGGGGGCCAGACCAGCAGAAGGCGTACCAG
GAGATTAAGCAGGCGCTGCTGACGGCCCCAGCGCTCGGCCTACCAGACCTGACGAAGCCGTTCGAGCTGTTCGTTG
ACGAGAAGCAGGGGTACGCGAAGGGCGTGCTGACACAGAAGCTGGGGCCTTGGCGCCGCCCGGTCGCGTACCTGT
CGAAGAAGCTGGACCCAGTCGCTGCTGGGTGGCCTCCATGCCTCCGGATGGTCGCTGCTATTGCGGTTCTGACCAA
GGATGCGGGGAAGCTCACAATGGGGCAGCCTCTCGTGATCCTGGCTCCACATGCGGTGGAGGCGCTGGTGAAGCA
GCCACCGGACCGGTGGCTGTCGAACGCTCGGATGACACACTACCAGGCGCTCCTCCTCGATACAGACCGGGTTCAG
TTCGGGCCTGTGGTTGCTCTGAACCCAGCCACACTGCTGCCACTCCCTGAGGAGGGCCTCCAGCACAATTGCCTCG
ACATCCTGGCTGAGGCGCACGGCACCCGCCCTGATCTCACCGACCAGCCTCTGCCAGATGCTGACCACACCTGGTA
CACGGATGGGTCCTCGCTGCTGCAGGAGGGCCAGAGGAAGGCGGGCGCCGCCGTCACCACAGAGACAGAGGTTAT
TTGGGCCAAGGCCCTACCGGCTGGCACCAGCGCCCAGCGCGCTGAGCTGATCGCGCTGACTCAGGCGCTGAAGAT
GGCCGAGGGGAAGAAGCTCAATGTTTACACCGACTCGCGGTACGCGTTCGCTACAGCTCACATTCATGGGGAGATCT
ACCGCCGGCGCGGGTGGCTGACTTCGGAGGGCAAGGAGATTAAGAATAAGGACGAGATCCTGGCCCTGCTCAAGG
CGCTGTTCCTGCCGAAGCGCCTCTCAATCATTCACTGCCCGGGCCACCAGAAGGGCCATTCGGCCGAGGCTAGGGG
CAATCGGATGGCTGACCAGGCGGCGCGGAAGGCGGCTATCACCGAGACTCCCGATACATCTACCCTCCTGATCGAG
AACTCGAGCCCATCTGGCGGCTCTAAGCGGACTGCGGATGGGTCTGAGTTCGAGTCACCAAAGAAGAAGAGGAAGG
TGGGCTCTGGCCCTGCTGCTAAGCGCGTGAAGCTCGATTGA

The background colors of the nucleotide sequence correspond to the functional elements shown above.
“//” indicates that the sequence is omitted for concision (see the full sequence in 492)

Supplementary Sequence S5. 4A96c (Csy4-P2A-PE6c)

Csy4-P2A-NLS-SpCas9KK-H840A-2xSGGS-NLS-2xSGGS-PE6cRT-SGGS- NLS-NLS$^{c\text{-}Myc}$

ATGGACCACTACCTGGACATCAGGCTCAGGCCGGACCCTGAGTTCCCGCCTGCCCAGCTGATGTCCGTGCTGTTCGG
CAAGCTGCATCAGGCTCTCGTTGCTCAGGGCGGCGATCGCATCGGCGTCTCATTCCCTGATCTGGACGAGTCTAGGA
GCAGGCTGGGCGAGAGGCTCCGCATCCATGCCAGCGCTGACGACCTGAGGGCTCTGCTCGCCAGGCCGTGGCTGG
AGGGCCTCAGGGACCATCTCCAGTTCGGCGAGCCTGCTGTTGTGCCACATCCAACACCGTACAGGCAGGTCAGCAG
GGTGCAGGCCAAGTCCAATCCTGAGAGGCTGCGCAGGCGCCTCATGAGGCGCCATGATCTGTCTGAGGAGGAGGCT
AGGAAGCGCATCCCTGATACCGTTGCTAGGGCGCTGGATCTGCCATTCGTCACACTGCGCTCACAGAGCACTGGCCA
GCACTTCAGGCTGTTCATCAGGCATGGCCCACTCCAGGTCACTGCTGAGGAGGGCGGCTTCACATGCTACGGCCTCT
CCAAGGGCGGCTTCGTTCCGTGGTTCGGCTCTGGCGCTACTAACTTCTCACTGCTGAAGCAGGCTGGCGATGTTGAG
GAGAATCCTGGCCCTATGAAGCGCACAGCCGATGGCAGCGAGTTCGAGTCACCTAAGAAGAAGCGCAAGGTGGAC//G
ACTCAGGCGGCTCATCTGGCGGGTCAAAGCGCACAGCCGACGGCTCTGAGTTCGAGAGCCCTAAGAAGAAGCGCAA
GGTGTCAGGCGGCTCTTCAGGCGGCAGCATCTCAAGCTCTAAGCACACGCTGTCGCAGATGAACAAGGTGTCGAAC
ATCGTCAAGGAGCCTGAGCTGCCGGACATCTACAAGGAGTTCAAGGACATCACCGCCGATACTAACACGGAGAAGCT
GCCCAAGCCAATCAAGGGCCTGGAGTTCGAGGTTGAGCTGACCCAGGAGAACTACCGCCTGCCCATCAGGAACTAC
CCACTCACACCGGTGAAGATGCAGGCGATGAATGATGAGATCAACCAGGGCCTCAAGGGCGGCATCATCCGCGAGT
CAAAGGCTATCAACGCCTGCCCGGTCATCTTCGTTCCACGCAAGGAGGGCACCCTGAGGATGGTGGTTGACTACAG
GCCACTGAACAAGTATGTCAAGCCAAACGTGTACCCGCTCCCGCTGATTGAGCAGCTGCTCGCCAAGATCCAGGGCT
CTACTATCTTCACGAAGCTCGATCTGAAGAGCGCTTACCACCAGATCCGGGTGCGCAAGGGCGATGAGCACAAGCTC
GCCTTCCGCTGCCCACGCGGCGTCTTCGAGTACCTCGTGATGCCGTACGGCATCAAGACAGCTCCAGCGCACTTCC
AGTACTTCATCAACACGATCCTGGGCGAGGCCAAGGAGTCGCATGTGGTGTGCTACATGGACGACATCCTGATTCAT
TCGAAGTCTGAGAGCGAGCACGTGAAGCACGTCAAGGATGTGCTGCAGAAGCTCAAGAATGCCAACCTCATCATCAA
TCAGGCGAAGTGCGAGTTCCACCAGTCTCAGGTCAAGTTCCTCGGCTACCACATCTCTGAGAAGGGCCTCACACCCT
GCCAGGAGAACATCGACAAGGTGCTCCAGTGGAAGCAGCCTAAGAATCAGAAGGAGCTGCGCCAGTTCCTCGGCCA
GGTGAACTACCTCCGCAAGTTCATTCCAAAGACTTCACAGCTGACGCACCCACTGAACAAGCTGCTCAAGAAGGATG
TGCGGTGGAAGTGGACTCCAACTCAGACGCAGGCTATCGAGAACATCAAGCAGTGCCTCGTGTCACCACCCGTGCT
GCGCCACTTCGACTTCAGCAAGAAGATCCTGCTGGAGACGGATGTGTCGGATGTCGCTGTCGGCGCTGTGCTCAGC
CAGAAGCACGACGATGACAAGTACTACCCGGTTGGCTACTACTCAGCGAAGATGAGCAAGGCCCAGCTCAACTACTC
TGTCTCAGACAAGGAGATGCTCGCTATCATCAAGTCACTCGAGCACTGGCGCCACTACCTCGAGTCAACTATCGAGC
CATTCAAGATCCTGACCGACCACAGGAATCTGATCGGCCGCATCACTAACGAGTCGGAGCCAGAGAACAAGCGGCTC
GCGAGGTGGCAGCTGTTCCTCCAGGACTTCAACTTCGAGATCAACTACAGGCCAGGCTCGGCGAATCACATCGCCG
ACGCCCTGTCACGCATTGTTGATGAGACAGAGCCGATTCCGAAGGACAACGAGGACAACAGCATCAACTTCGTCAAC
CAGATCAGCATCTCTGGCGGCTCTAAGCGGACTGCGGATGGGTCTGAGTTCGAGTCACCAAAGAAGAAGAGGAAGG
TGGGCTCTGGCCCTGCTGCTAAGCGCGTGAAGCTCGATTGA

The background colors of the nucleotide sequence correspond to the functional elements shown above. "//" indicates that the sequence is omitted for concision (see the full sequence in 492)

Supplementary Sequence S6. 4A96d (Csy4-P2A-PE6d)

Csy4-P2A-NLS-SpCas9KK-H840A-2xSGGS-NLS-2xSGGS-PE6dRT-SGGS- NLS-NLS$^{c\text{-}Myc}$

ATGGACCACTACCTGGACATCAGGCTCAGGCCGGACCCTGAGTTCCCGCCTGCCCAGCTGATGTCCGTGCTGTTCGG
CAAGCTGCATCAGGCTCTCGTTGCTCAGGGCGGCGATCGCATCGGCGTCTCATTCCCTGATCTGGACGAGTCTAGGA
GCAGGCTGGGCGAGAGGCTCCGCATCCATGCCAGCGCTGACGACCTGAGGGCTCTGCTCGCCAGGCCGTGGCTGG
AGGGCCTCAGGGACCATCTCCAGTTCGGCGAGCCTGCTGTTGTGCCACATCCAACACCGTACAGGCAGGTCAGCAG
GGTGCAGGCCAAGTCCAATCCTGAGAGGCTGCGCAGGCGCCTCATGAGGCGCCATGATCTGTCTGAGGAGGAGGCT
AGGAAGCGCATCCCTGATACCGTTGCTAGGGCGCTGGATCTGCCATTCGTCACACTGCGCTCACAGAGCACTGGCCA
GCACTTCAGGCTGTTCATCAGGCATGGCCCACTCCAGGTCACTGCTGAGGAGGGCGGCTTCACATGCTACGGCCTCT
CCAAGGGCGGCTTCGTTCCGTGGTTCGGCTCTGGCGCTACTAACTTCTCACTGCTGAAGCAGGCTGGCGATGTTGAG
GAGAATCCTGGCCCTATGAAGCGCACAGCCGATGGCAGCGAGTTCGAGTCACCTAAGAAGAAGCGCAAGGTGGAC//G
ACTCAGGCGGCTCATCTGGCGGGTCAAAGCGCACAGCCGACGGCTCTGAGTTCGAGAGCCCTAAGAAGAAGCGCAA
GGTGTCAGGCGGCTCTTCAGGCGGCAGCACACTGAACATTGAGGATGAGTACAGGCTGCACGAGACTAGCAAGGAG
CCAGACGTTAGCCTCGGCAGCACATGGCTCTCTGACTTCCCACAGGCTTGGGCCGAGACTGGCGGCATGGGCCTGG
CCGTGAGGCAGGCTCCACTGATCATCCCTCTGAAGGCCACCAGCACACCTGTTAGCATCAAGCAGTACCCTATGTCA
CAGGAGGCCAGGCTCGGCATCAAGCCACACATTCAGCGGCTGCTGGACCAGGGCATCCTGGTGCCATGCCAGTCAC
CTTGGAACACACCACTCCTGCCTGTCAAGAAGCCTGGGACTAACGACTACAGGCCCGTTCAGGATCTCAGGGAGGTC
AACAAGCGCGTTGAGGACATCCATCCCAATGTGCCCAATCCGTACAATCTGCTGTCTGGCCTGCCTCCGTCACACCA
GTGGTACACTGTCCTGGACCTCAAGGATGCGTTCTTCTGCCTGCGGCTGCACCCGACATCACAGCCGCTGTTCGCGT
TCGAGTGGCGCGATCCAGAGATGGGCATCAGCGGCCAGCTGACATGGACACGCCTGCCACAGGGCTTCAAGAACTC
ACCGACACTGTTCTGCGAGGCTCTCCACAGGGATCTCGCTGACTTCAGGATTCAGCATCCCGATCTGATCCTGCTCC
AGTACTACGATGACCTGCTCCTGGCTGCTACATCTGAGCTGGACTGCCAGCAGGGCACACGCGCTCTGCTGCAGAC
ACTGGGCAATCTCGGCTACAGGGCCAGCGCTAAGAAGGCGCAGATCTGCCAGAAGCAGGTCAAGTACCTGGGCTAC
CTGCTCAAGGAGGGCCAGCGCTGGCTCACTGAGGCTCGCAAGGAGACTGTGATGGGCCAGCCTACACCTAAGACAC
CCAGGCAGCTCAGGGAGTTCCTCGGCACTGCTGGGTTCTGCAGGCTGTGGATCCCTGGGTTCGCTGAGATGGCTGC
GCCACTCTACCCACTGACTAAGACTGGCACACTGTTCAACTGGGGCCCAGATCAGCAGAAGGCGTACCAGGAGATCA
AGCAGGCGCTGCTGACTGCTCCTGCGCTCGGCCTGCCTGATCTGACCAAGCCATTCGAGCTGTTCGTTGATGAGAAG
CAGGGCTACGCTAAGGGCGTGCTGACACAGAAGCTCGGGCCTTGGCGCAGGCCAGTCGCGTACCTGTCCAAGAAG
CTCGATCCAGTCGCTGCTGGGTGGCCACCATGCCTCAGGATGGTCGCTGCTATCGCCGTTCTGACTAAGGATGCTG
GCAAGCTCACTATGGGCCAGCCACTCGTGATCCTGGCTCCACACGCCGTGGAGGCGCTGGTGAAGCAGCCACCTGA
TAGGTGGCTGTCTAACGCTAGGATGACACACTACCAGGCGCTGCTGCTCGATACTGATAGGGTTCAGTTCGGGCCTG
TGGTTGCTCTGAATCCAGCCACACTGCTGCCACTCCCTGAGGAGGGCCTCCAGCACAACTGCCTCTCTGGCGGCTCT
AAGCGGACTGCGGATGGGTCTGAGTTCGAGTCACCAAAGAAGAAGAGGAAGGTGGGCTCTGGCCCTGCTGCTAAGC
GCGTGAAGCTCGATTGA

The background colors of the nucleotide sequence correspond to the functional elements shown above. "//" indicates that the sequence is omitted for concision (see the full sequence in 492)

Supplementary Sequence S7. RNA cassette V1

35S-CmYLCV-U6-Csy4RS-Guide-sgRNAm-rtT-PBS-linker-evopreQ1-Csy4RS-Guide-sgRNAm2-Csy4RS-polyT-HSPt

ATGGAGTCAAAGATTCAAATAGAGGACCTAACAGAACTCGCCGTAAAGACTGGCGAACAGTTCATACAGAGTCTCTTAC
GACTCAATGACAAGAAGAAAATCTTCGTCAACATGGTGGAGCACGACACACTTGTCTACTCCAAAAATATCAAAGATAC
AGTCTCAGAAGACCAAAGGGCAATTGAGACTTTTCAACAAAGGGTAATATCCGGAAACCTCCTCGGATTCCATTGCCCA
GCTATCTGTCACTTTATTGTGAAGATAGTGGAAAAGGAAGGTGGCTCCTACAAATGCCATCATTGCGATAAAGGAAAGG
CCATCGTTGAAGATGCCTCTGCCGACAGTGGTCCCAAAGATGGACCCCCACCCACGAGGAGCATCGTGGAAAAAGAA
GACGTTCCAACCACGTCTTCAAAGCAAGTGGATTGATGTGATTGGCAGACATACTGTCCCACAAATGAAGATGGAATCT
GTAAAAGAAAACGCGTGAAATAATGCGTCTGACAAAGGTTAGGTCGGCTGCCTTTAATCAATACCAAAGTGGTCCCTAC
CACGATGGAAAAACTGTGCAGTCGGTTTGGCTTTTTCTGACGAACAAATAAGATTCGTGGCCGACAGGTGGGGGTCCA
CCATGTGAAGGCATCTTCAGACTCCAATAATGGAGCAATGACGTAAGGGCTTACGAAATAAGTAAGGGTAGTTTGGGAA
ATGTCCACTCACCCGTCAGTCTATAAATACTTAGCCCCTCCCTCATTGTTAAGGGAGCAAAATCTCAGAGAGATAGTCCT
AGAGAGAGAAAGAGAGCAAGTAGCCTAGAAGTAGTCAAGGCGGCGAAGTATTCAGGCACGTGGCCAGGAAGAAGAAA
AGCCAAGACGACGAAAACAGGTAAGAGCTAAGCATCTAGGTAAGTTGAAAACAATCTTCAAAAGTCCCACATCGCTTAG
ATAAGAAAACGAAGCTGAGTTTATATACAGCTAGAGTCGAAGTAGTGATTGGTTCACTGCCGTATAGGCAGNNNNNNNN
NNNNNNNNNNNNGTTTAAGAGCTATGCTGGAAACAGCATAGCAAGTTTAAATAAGGCTAGTCCGTTATCAACTTGAAAA
AGTGGCACCGAGTCGGTGCNNNNNNNNNNNNNNNNNNNNNNNNNNNNNNNNNNNNNCGCGGTTCTATCTAGTTACG
CGTTAAACCAACTAGAAAGTTCACTGCCGTATAGGCAGNNNNNNNNNNNNNNNNNNNNGTTTCAGAGCTATGCTGGAA
ACAGCATAGCAAGTTGAAATAAGGCTAGTCCGTTATCAACTTGAAAAAGTGGCACCGAGTCGGTGCGTTCACTGCCGT
ATAGGCAGTTTTTTTTGATATCTCCGGGGCTAATTGAATATGAAGATGAAGATGAAATATTTGGTGTGTCAAATAAAAAGC
TGGTGTGCTTAAGTTTGTGTTTTTTTCTTGGCTTGTTGTGTTATGAATTTGTGGCTTTTTCTAATATTAAATGAATGTAAGA
TCTCATTATAATGAATAAACAAATGTTTCTATAATCCATTGTGAATGTTTTGTTGGATCTCTTCTGCAGCATATAACTACTGT
ATGTGCTATGGTATGGACTATGGAATATGATTAAAGATAA

The background colors of the nucleotide sequence correspond to the functional elements shown above.

Supplementary Sequence S8. RNA cassette V2

Cloning cassette in RNA-V2 which include two epegRNAs and one nicking sgRNA for one target.

35S-CmYLCV-U6-Csy4RS-Guide-sgRNAm-rtT-PBS-linker-evopreQ1-Csy4RS-Guide-sgRNAm-rtT-PBS-linker-evopreQ1-Csy4RS-Guide-sgRNAm2-Csy4RS-polyT-HSPt

ATGGAGTCAAAGATTCAAATAGAGGACCTAACAGAACTCGCCGTAAAGACTGGCGAACAGTTCATACAGAGTCTCTTAC
GACTCAATGACAAGAAGAAAATCTTCGTCAACATGGTGGAGCACGACACACTTGTCTACTCCAAAAATATCAAAGATAC
AGTCTCAGAAGACCAAAGGGCAATTGAGACTTTTCAACAAAGGGTAATATCCGGAAACCTCCTCGGATTCCATTGCCCA
GCTATCTGTCACTTTATTGTGAAGATAGTGGAAAAGGAAGGTGGCTCCTACAAATGCCATCATTGCGATAAAGGAAAGG
CCATCGTTGAAGATGCCTCTGCCGACAGTGGTCCCAAAGATGGACCCCCACCCACGAGGAGCATCGTGGAAAAAGAA
GACGTTCCAACCACGTCTTCAAAGCAAGTGGATTGATGTGATTGGCAGACATACTGTCCCACAAATGAAGATGGAATCT
GTAAAAGAAAACGCGTGAAATAATGCGTCTGACAAAGGTTAGGTCGGCTGCCTTTAATCAATACCAAAGTGGTCCCTAC
CACGATGGAAAAACTGTGCAGTCGGTTTGGCTTTTTCTGACGAACAAATAAGATTCGTGGCCGACAGGTGGGGGTCCA
CCATGTGAAGGCATCTTCAGACTCCAATAATGGAGCAATGACGTAAGGGCTTACGAAATAAGTAAGGGTAGTTTGGGAA
ATGTCCACTCACCCGTCAGTCTATAAATACTTAGCCCCTCCCTCATTGTTAAGGGAGCAAAATCTCAGAGAGATAGTCCT
AGAGAGAGAAAGAGAGCAAGTAGCCTAGAAGTAGTCAAGGCGGCGAAGTATTCAGGCACGTGGCCAGGAAGAAGAAA
AGCCAAGACGACGAAAACAGGTAAGAGCTAAGCATCTAGGTAAGTTGAAAACAATCTTCAAAAGTCCCACATCGCTTAG
ATAAGAAAACGAAGCTGAGTTTATATACAGCTAGAGTCGAAGTAGTGATTgGTTCACTGCCGTATAGGCAGNNNNNNNN
NNNNNNNNNNNNGTTTAAGAGCTATGCTGGAAACAGCATAGCAAGTTTAAATAAGGCTAGTCCGTTATCAACTTGAAAA
AGTGGCACCGAGTCGGTGCNNNNNNNNNNNNNNNNNNNNNNNNNNNNNNNNNNNNNCGCGGTTCTATCTAGTTACG
CGTTAAACCAACTAGAAAGTTCACTGCCGTATAGGCAGNNNNNNNNNNNNNNNNNNNNGTTTAAGAGCTATGCTGGAA
ACAGCATAGCAAGTTTAAATAAGGCTAGTCCGTTATCAACTTGAAAAAGTGGCACCGAGTCGGTGCNNNNNNNNNNNN
NNNNNNNNNNNNNNNNNNNNNNNNNCGCGGTTCTATCTAGTTACGCGTTAAACCAACTAGAAAGTTCACTGCCGTATA
GGCAGNNNNNNNNNNNNNNNNNNNNGTTTCAGAGCTATGCTGGAAACAGCATAGCAAGTTGAAATAAGGCTAGTCCG
TTATCAACTTGAAAAAGTGGCACCGAGTCGGTGCGTTCACTGCCGTATAGGCAGTTTTTTTTGATATCTCCGGGGCTAA
TTGAATATGAAGATGAAGATGAAATATTTGGTGTGTCAAATAAAAAGCTGGTGTGCTTAAGTTTGTGTTTTTTTCTTGGCT
TGTTGTGTTATGAATTTGTGGCTTTTTCTAATATTAAATGAATGTAAGATCTCATTATAATGAATAAACAAATGTTTCTATAAT
CCATTGTGAATGTTTTGTTGGATCTCTTCTGCAGCATATAACTACTGTATGTGCTATGGTATGGACTATGGAATATGATTAA
AGATAA

The background colors of the nucleotide sequence correspond to the functional elements shown above.

Supplementary Sequence S9. RNA cassette V1×2

35S-CmYLCV-U6-Csy4RS-Guide-sgRNAm-rtT-PBS-linker-evopreQ1-Csy4RS-Guide-sgRNAm-rtT-PBS-linker-evopreQ1-Csy4RS-Guide-sgRNAm2-Csy4RS-Guide-sgRNAm2-Csy4RS-polyT-HSPt

ATGGAGTCAAAGATTCAAATAGAGGACCTAACAGAACTCGCCGTAAAGACTGGCGAACAGTTCATACAGAGTCTCTTAC
GACTCAATGACAAGAAGAAAATCTTCGTCAACATGGTGGAGCACGACACACTTGTCTACTCCAAAAATATCAAAGATAC
AGTCTCAGAAGACCAAAGGGCAATTGAGACTTTTCAACAAAGGGTAATATCCGGAAACCTCCTCGGATTCCATTGCCCA
GCTATCTGTCACTTTATTGTGAAGATAGTGGAAAAGGAAGGTGGCTCCTACAAATGCCATCATTGCGATAAAGGAAAGG
CCATCGTTGAAGATGCCTCTGCCGACAGTGGTCCCAAAGATGGACCCCCACCCACGAGGAGCATCGTGGAAAAAGAA
GACGTTCCAACCACGTCTTCAAAGCAAGTGGATTGATGTGATTGGCAGACATACTGTCCCACAAATGAAGATGGAATCT
GTAAAAGAAAACGCGTGAAATAATGCGTCTGACAAAGGTTAGGTCGGCTGCCTTTAATCAATACCAAAGTGGTCCCTAC
CACGATGGAAAAACTGTGCAGTCGGTTTGGCTTTTTCTGACGAACAAATAAGATTCGTGGCCGACAGGTGGGGGTCCA
CCATGTGAAGGCATCTTCAGACTCCAATAATGGAGCAATGACGTAAGGGCTTACGAAATAAGTAAGGGTAGTTTGGGAA
ATGTCCACTCACCCGTCAGTCTATAAATACTTAGCCCCTCCCTCATTGTTAAGGGAGCAAAATCTCAGAGAGATAGTCCT
AGAGAGAGAAAGAGAGCAAGTAGCCTAGAAGTAGTCAAGGCGGCGAAGTATTCAGGCACGTGGCCAGGAAGAAGAAA
AGCCAAGACGACGAAAACAGGTAAGAGCTAAGCATCTAGGTAAGTTGAAAACAATCTTCAAAAGTCCCACATCGCTTAG
ATAAGAAAACGAAGCTGAGTTTATATACAGCTAGAGTCGAAGTAGTGATTgGTTCACTGCCGTATAGGCAGNNNNNNNN
NNNNNNNNNNNNGTTTAAGAGCTATGCTGGAAACAGCATAGCAAGTTTAAATAAGGCTAGTCCGTTATCAACTTGAAAA
AGTGGCACCGAGTCGGTGCNNNNNNNNNNNNNNNNNNNNNNNNNNNNNNNNNNNNNCGCGGTTCTATCTAGTTACG
CGTTAAACCAACTAGAAAGTTCACTGCCGTATAGGCAGNNNNNNNNNNNNNNNNNNNNGTTTAAGAGCTATGCTGGAA
ACAGCATAGCAAGTTTAAATAAGGCTAGTCCGTTATCAACTTGAAAAAGTGGCACCGAGTCGGTGCNNNNNNNNNNNN
NNNNNNNNNNNNNNNNNNNNNNNNNCGCGGTTCTATCTAGTTACGCGTTAAACCAACTAGAAAGTTCACTGCCGTATA
GGCAGNNNNNNNNNNNNNNNNNNNNGTTTCAGAGCTATGCTGGAAACAGCATAGCAAGTTGAAATAAGGCTAGTCCG
TTATCAACTTGAAAAAGTGGCACCGAGTCGGTGCGTTCACTGCCGTATAGGCAGNNNNNNNNNNNNNNNNNNNNGTT
TCAGAGCTATGCTGGAAACAGCATAGCAAGTTGAAATAAGGCTAGTCCGTTATCAACTTGAAAAAGTGGCACCGAGTCG
GTGCGTTCACTGCCGTATAGGCAGTTTTTTTTGATATCTCCGGGGCTAATTGAATATGAAGATGAAGATGAAATATTTGGT
GTGTCAAATAAAAAGCTGGTGTGCTTAAGTTTGTGTTTTTTTCTTGGCTTGTTGTGTTATGAATTTGTGGCTTTTTCTAAT
ATTAAATGAATGTAAGATCTCATTATAATGAATAAACAAATGTTTCTATAATCCATTGTGAATGTTTTGTTGGATCTCTTCTG
CAGCATATAACTACTGTATGTGCTATGGTATGGACTATGGAATATGATTAAAGATAA

The background colors of the nucleotide sequence correspond to the functional elements shown above.
